# Trapped interfacial redox introduces reversibility in the oxygen reduction reaction in a non-aqueous Ca^2+^ electrolyte†

**DOI:** 10.1039/d0sc06991d

**Published:** 2021-05-28

**Authors:** Yi-Ting Lu, Alex R. Neale, Chi-Chang Hu, Laurence J. Hardwick

**Affiliations:** Stephenson Institute for Renewable Energy, Department of Chemistry, University of Liverpool Liverpool L69 7ZD UK hardwick@liverpool.ac.uk; Department of Chemical Engineering, National Tsing Hua University Hsin-Chu 300044 Taiwan cchu@che.nthu.edu.tw

## Abstract

Electrochemical investigations of the oxygen reduction reaction (ORR) and oxygen evolution reaction (OER) have been conducted in a Ca^2+^-containing dimethyl sulfoxide electrolyte. While the ORR appears irreversible, the introduction of a tetrabutylammonium perchlorate (TBAClO_4_) co-salt in excess concentrations results in the gradual appearance of a quasi-reversible OER process. Combining the results of systematic cyclic voltammetry investigations, the degree of reversibility depends on the ion pair competition between Ca^2+^ and TBA^+^ cations to interact with generated superoxide (O_2_^−^). When TBA^+^ is in larger concentrations, and large reductive overpotentials are applied, a quasi-reversible OER peak emerges with repeated cycling (characteristic of formulations without Ca^2+^ cations). *In situ* Raman microscopy and rotating ring-disc electrode (RRDE) experiments revealed more about the nature of species formed at the electrode surface and indicated the progressive evolution of a charge storage mechanism based upon *trapped interfacial redox*. The first electrochemical step involves generation of O_2_^−^, followed primarily by partial passivation of the surface by Ca_*x*_O_*y*_ product formation (the dominant initial reaction). Once this product matrix develops, the subsequent formation of TBA^+^--O_2_^−^ is contained within the Ca_*x*_O_*y*_ product interlayer at the electrode surface and, consequently, undergoes a facile oxidation reaction to regenerate O_2_.

## Abstract 2

An interlayer product of oxygen reduction with Ca^2+^/TBA^+^ yields a quasi-reversible oxygen evolution reaction by inducing a *trapped interfacial redox* process.

## Graphical abstract

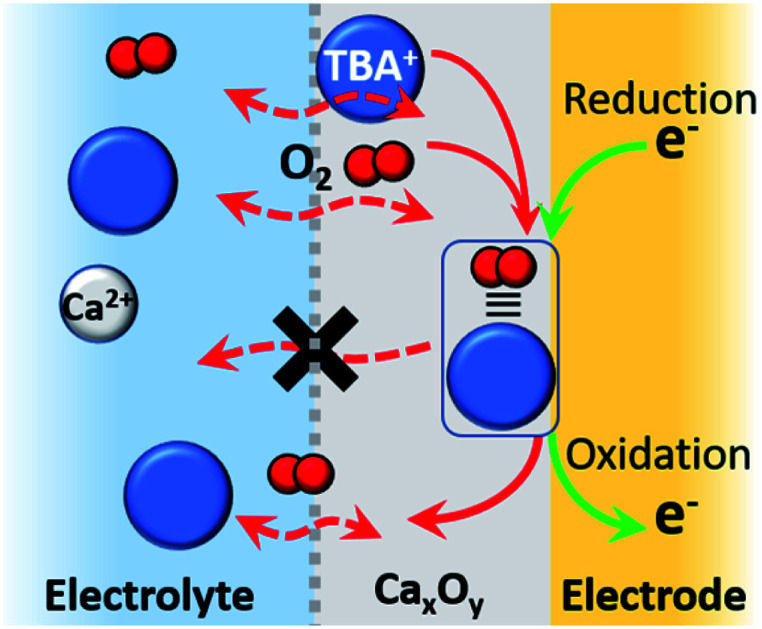

## 1. Introduction

Metal–air batteries are a promising candidate for energy storage applications because of their high theoretical specific energy compared to Li-ion batteries.^1^ So far, research attention has been mostly focused on Li–air batteries since Abraham and Jiang reintroduced the concept of Li–air cell using polymer electrolytes.^2^ More recently, research on Mg metal and its application in metal–air batteries have started to receive attention, by reason of its high volumetric capacity (3832 mA h cm^−3^ for Mg *vs.* 2062 mA h cm^−3^ for Li), reduced dendrite formation, sufficient natural abundance, environmental friendliness, and low cost.^3,4^ However, its analogue Ca (2073 mA h cm^−3^) has so far received less attention than Mg even though Ca possesses similar advantages to Mg.

For metal–air batteries, the oxygen reduction reaction (ORR) and oxygen evolution reaction (OER) on the air electrode side are of great importance. Taking the Li–air battery as an example, many researchers have made contributions to mechanistic investigations and fundamental studies, attempting to understand the underlying (electro)chemistry of ORR and OER in non-aqueous electrolytes. Abraham proposed to explain the ORR behaviour in aprotic electrolytes based on the hard–soft acid–base theory (HSAB).^5^ Bruce's group studied the influences of various solvents, which have different donor numbers, on the reaction pathway of ORR, and they suggested that LiO_2_ solubility in the solvent significantly affects the product distribution on the air electrode.^6^ However, unlike the Li–O_2_ electrochemistry, there is a limited knowledge of ORR/OER mechanisms in electrolyte systems containing divalent alkaline-earth cations (namely Mg^2+^ and Ca^2+^). With much less reversible oxygen electrochemistry in electrolytes containing Mg^2+^/Ca^2+^, some papers reported the use of redox mediators,^7–9^ which have already demonstrated more reversible cycling performance in Li–air cells.^10–13^ However, only few papers aimed to study the fundamental oxygen electrochemistry in electrolytes containing Ca^2+^. Recently, Baltruschat's group studied the ORR/OER electrochemistry in 0.4 M Ca^2+^-containing dimethyl sulfoxide (DMSO) with differential electrochemical mass spectrometry (DEMS).^14^ The obtained DEMS results show that peroxide species (O_2_^2−^) form during the ORR at Au electrodes, while superoxide species (O_2_^−^) form on Rh, Pt, Ru, and glassy carbon (GC) electrodes. On GC electrodes the amount of superoxide that can be re-oxidised on the positive potential sweep takes up *ca.* 95% of the reduced oxygen. The same group published a study on the ORR in DMSO containing Mg^2+^ and Ca^2+^ (also including Sr^2+^ and Ba^2+^), where DEMS results show peroxide species forms exclusively in the presence of Mg^2+^, and superoxide dominates in the presence of Ca^2+^.^15^ Generally speaking, the ORR/OER mechanism in aprotic electrolytes in the existence of Mg^2+^/Ca^2+^ remains in the early stages of investigation, and the oxygen electrochemistry requires further exploration in a wide range of electrolyte formulations.

This work reports on the electrochemistry of ORR/OER in DMSO-based electrolytes containing Ca^2+^ in which the tetrabutylammonium (TBA^+^) cation is also present as a co-salt. Such experiments have been conducted in Li–O_2_ systems,^6,16^ but not yet explored in Ca^2+^-based electrolytes. A series of electrochemical tests with various applied potentials and salt concentrations were carried out to characterise the ORR/OER, which revealed a charge storage mechanism resulting from TBA^+^--O_2_^−^ being contained within a Ca_*x*_O_*y*_ interlayer at the electrode surface.

## 2. Experimental

### 2.1 Chemicals

Calcium perchlorate (Ca(ClO_4_)_2_) and tetrabutylammonium perchlorate (TBAClO_4_) (Sigma-Aldrich) were dried under vacuum overnight at 90 °C. DMSO (ROMIL, UK) was dried with freshly activated molecular sieves (4 Å) (Alfa Aesar) for 1 week prior to use. CaO_2_ (75% purity), Ca(OH)_2_, and CaO (Sigma-Aldrich) were dried under vacuum overnight at 70 °C prior to Raman measurements.

### 2.2 Electrochemical measurements

Electrochemical measurements were performed using VSP and SP-150 potentiostat workstations (BioLogic, France) inside an Ar-filled glovebox (<0.1 ppm H_2_O, 0.1 ppm O_2_). A previously reported airtight cell, which allows a gas inlet and outlet for bubbling the electrolyte with oxygen, was applied.^17^ Prior to each experiment, the electrolyte was purged with Ar for 30 min to remove any possible impurity gases, and the electrolyte was then bubbled with O_2_ (99.9995%, further dried through a P_2_O_5_ tube) for 30 min to saturate the electrolyte. Dry Ar and O_2_ gas lines were used to purge and bubble electrolytes, with water contents below 20 ppm, determined by Karl Fischer titration after the gas bubbling. Cyclic voltammetry (CV) was conducted at the scan rate of 100 mV s^−1^ (unless otherwise stated) on gold disc electrodes (ALS, Japan) with a diameter of 3 mm. Supporting CV experiments were also conducted with glassy carbon (GC) and Pt disc electrodes (ALS, Japan). A silver wire was used as the quasi-reference electrode and a platinum wire was used as the counter electrode. The reference electrode was calibrated by ferrocene/ferrocenium couple in DMSO (0.67 V *vs.* standard hydrogen electrode (SHE)^18^) and reported with respect to Ag^+^/Ag couple in DMSO (0.49 V *vs.* SHE^19^). The rotating ring-disc electrode (RRDE) experiments were carried out using a GC disc and Pt ring electrode, wherein the rotation was controlled with an MSR electrode rotator (PINE, USA) in combination with the VSP potentiostat described above. All working electrodes were carefully polished using Al_2_O_3_ (1.0 μm, 0.3 μm, and 0.05 μm) slurries and dried overnight in vacuum at 100 °C before use. All glassware was dried under the same condition prior to use.

### 2.3 Electrochemical surface-enhanced Raman spectroscopy

Raman spectra were recorded on an In-Via Raman spectrometer (Renishaw, UK) with an inverted microscope (Leica) using a 633 nm laser. An airtight glass cell with a sapphire window at the bottom for the incident laser was used for Raman measurements. The Au working electrode was roughened following previously reported procedures prior to the surface-enhanced Raman spectroscopy (SERS) measurements.^20^ The glass cell was bubbled with O_2_ for 30 min and then sealed completely before being transferred to the spectrometer. The SP-150 potentiostat was used to control the applied potential during the electrochemical SERS measurements.

## 3. Results and discussion

### 3.1 Electrochemical investigations of the oxygen electrochemistry

**Fig. 1:**
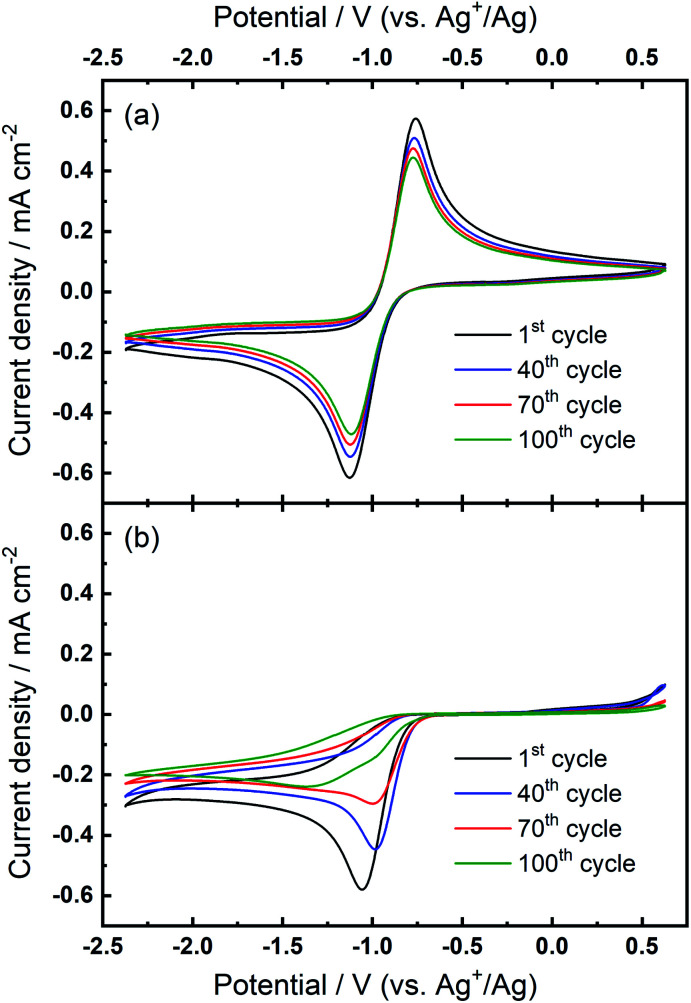
(a) Cyclic voltammograms of the ORR and OER on an Au electrode at 100 mV s^−1^ in O_2_-saturated (a) 0.1 M TBAClO_4_/DMSO and (b) 0.1 M Ca(ClO_4_)_2_/DMSO.

CV was applied at a planar Au disc electrode to characterise the ORR and OER in O_2_-saturated DMSO electrolytes containing 0.1 M of either TBAClO_4_ or Ca(ClO_4_)_2_ salt (Fig. 1). Fig. 1(a) displays a typical reversible one-electron reduction/oxidation in the presence of TBAClO_4_, where the free superoxide (or weakly coordinated superoxide, O_2_^−^) is generated, as reported previously.^5,21,22^ The onset potential of reduction occurs at *ca.* −0.87 V (*vs.* Ag^+^/Ag), with the peak currents for ORR and OER at potentials of −1.12 V and −0.76 V, respectively. In contrast, the irreversible CV in the presence of Ca(ClO_4_)_2_ is observed (Fig. 1(b)). Reduction current responses are observed (onset potential at *ca.* −0.81 V), yet no reversible oxidation current occurs on the reverse sweep, apart from a small current rise at potentials over 0.48 V. Similar CVs without reversibility have been reported using Au electrodes in the presence of divalent Ca^2+^ (and Mg^2+^) cations.^14,15,23,24^ The lack of oxygen evolution indicates the reaction product, which was reported to be a peroxy species by DEMS,^14,15^ cannot be readily re-oxidised in the investigated potential window.

Over numerous potential sweeps, the oxygen reduction peak in the Ca^2+^-containing electrolyte becomes distorted, indicating the formation and growth of a blocking layer on the electrode surface. However, since the reduction current response persists after 100 cycles, it can be inferred that the surface does not become completely passivated and insulated by the ORR product(s). The moderate degree of passivation in this Ca–O_2_ system is distinct from the analogous Li–O_2_ system where the ORR current usually shows a large hysteresis loop on the reverse scan.^25–29^ Pt and GC electrodes with the same geometric area were also tested in the same electrolyte for comparison (see Fig. S1 and S2 in the ESI†). In spite of some differences in shape between CVs measured on different electrodes (see corresponding discussion in the ESI†), the oxygen reduction within the studied range in Ca^2+^-containing electrolyte is generally irreversible, independent of electrode substrate, with only minor oxidation currents measured in the region of 0–0.5 V. However, despite important experimental differences, the irreversibility of the ORR/OER in Fig. S1 and S2† is different from the result reported by Reinsberg *et al.*^14^ In that work, critically, the authors utilised a dual thin-layer cell under forced electrolyte convection, slower voltage scan rates (10 mV s^−1^), greater Ca-salt concentrations (0.1–0.4 M), and lower O_2_ concentrations (20% O_2_ in Ar). Therein, the authors observed a small but distinct oxidation peak for the ORR product, identified as superoxide by DEMS, on Pt and GC when a high oxidation overpotential was applied (*ca.* +0.3 V *vs.* Ag^+^/Ag). Conversely, while oxidation currents occur from 0–0.5 V only in the first cycles in Fig. 1(b), S1 and S2,† these are comparatively low (relative to peak currents for reduction) and diminish further with progressive cycling. Only the Pt electrode (Fig. S1†) displays some evidence of peak formation at 0–0.1 V in the first cycles, and any such residual currents could be attributed to oxidation of calcium oxide (Ca_*x*_O_*y*_) species formed during the first cycles. The key difference here could relate to large reductive overpotentials on the negative sweep, driving successive reactions of superoxide species and, thus, reducing apparent reversibility when compared to Reinsberg *et al.*^14^ Critically though, the more negative reduction potentials utilised here (−2.37 V) would not be expected to exceed the reductive stability limit for potential-induced decomposition of DMSO. To validate this, the electrochemical window of 0.1 M TBAClO_4_/DMSO at Au and GC (presented in Fig. S3 and S4,† respectively) was measured by cyclic voltammetry and the onset for reductive decomposition was estimated at −2.7 to −2.8 V. The long-term instability of DMSO with respect to superoxide radical intermediates has also been reported.^30^ This is an important consideration for long-term application in full metal–air cells, but is not expected to present prominent issues on the timescales of investigations discussed here. The explanation for the differences observed in the reversibility of the ORR will be discussed and explored further with our proposed mechanism at a later stage within the manuscript.

**Fig. 2:**
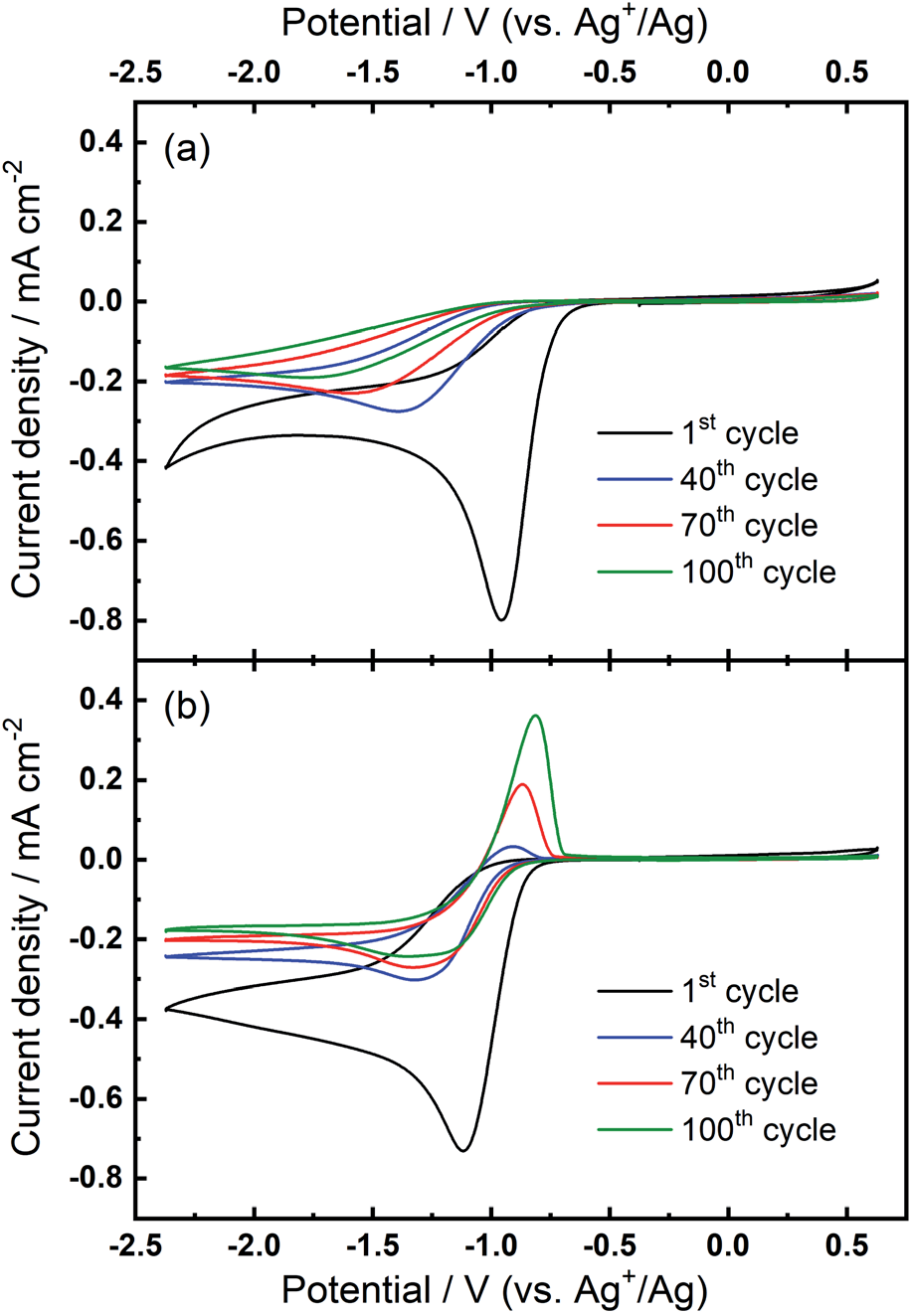
Cyclic voltammograms of the ORR and OER on Au at 100 mV s^−1^ in O_2_-saturated (a) 0.1 M TBAClO_4_/0.1 M Ca(ClO_4_)_2_/DMSO and (b) 0.1 M TBAClO_4_/0.01 M Ca(ClO_4_)_2_/DMSO.

For the investigation of ORR/OER in non-aqueous metal–air battery electrolytes containing both quaternary ammonium and alkali metal cations, the ammonium salt would largely be introduced as a supporting electrolyte to increase the ionic conductivity of the electrolyte solution.^20,31–33^ In other words, the alkali metal cation generally dominates the electrochemical reactions and the electrochemistry is expected to be less influenced by the supporting ammonium salt that may generally only experience weak coordination interactions with superoxide anions owing to the bulky structure of soft cation. Fig. 2(a) shows that the combination of both 0.1 M TBAClO_4_ and 0.1 M Ca(ClO_4_)_2_ does not lead to any electrochemical reversibility of the ORR/OER, which seems consistent with the fact that TBAClO_4_ merely contributes to ionic conductivity. However, Fig. 2(b) displays an interesting feature in the CV. When the TBA^+^ has a much higher concentration (0.1 M) than Ca^2+^ (0.01 M), an oxidation peak centred at *ca.* −0.87 V appears after a few CV cycles. Above −0.7 V, no apparent anodic current can be seen. Although the reversibility in terms of the respective peak current densities of reduction and oxidation is low compared with that in Fig. 1(a) where Ca^2+^ is not present, the peak separation between the reduction peak and the newly growing oxidation peak in Fig. 2(b) is rather close. The position of this OER peak is indicative of a low overpotential for oxidation of this ORR intermediate/product. However, it is interesting to note the OER peak shapes for Ca^2+^-containing electrolyte differs significantly from that containing only TBA^+^. A direct comparison of the peak shapes is presented in Fig. S5.† Therein, the OER current for the Ca^2+^-containing electrolyte displays a non-Cottrellian current decay after the peak and the current decays rapidly after the peak to nearly zero, indicative of a strong deviation away from kinetics controlled by diffusion of the reactant species.

Similar phenomena relating to the evolution of a new OER peak were also observed with continued cycling at both GC and Pt electrodes within the same electrolyte formulations (see Fig. S6 in the ESI†). While the evolution of both OER and ORR shows some interesting differences in the CVs at Au, GC, and Pt, quantification of the differences in electrocatalytic activity would be valuable but lie beyond the scope of the present study. Critically to this investigation, however, the evolution of this quasi-reversible OER response with the characteristic non-Cottrellian-like peak shape (described above) is observed for all three substrates. Furthermore, we demonstrated the formation of the quasi-reversible OER peak is also observed when the TBA^+^ salt is substituted with the smaller tetraethylammonium (TEA^+^) analogue. The cyclic voltammograms of O_2_-saturated electrolyte containing 0.1 M TEAClO_4_/0.01 M Ca(ClO_4_)_2_/DMSO at Au provided in Fig. S7† show the formation and growth of a quasi-reversible OER peak of similar potential and shape. Further characterisation of the effects of different co-salts (and solvents) are the subject of continued investigations but this observation highlights the observed effect is not exclusive to the combined system of TBA^+^ and Ca^2+^.

**Fig. 3:**
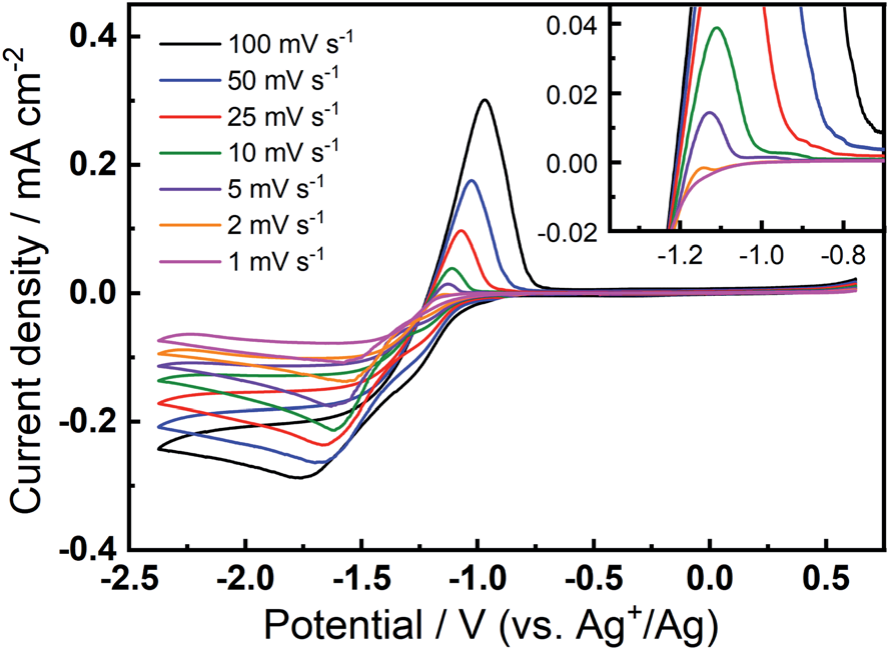
Cyclic voltammograms of the ORR and OER on Au at various scan rates in O_2_-saturated 0.1 M TBAClO_4_/0.01 M Ca(ClO_4_)_2_/DMSO. The inset shows the enlarged positive sweeps to show the OER features at lower scan rates. Variable scan rate voltammograms were collected after 150 cycles at 100 mV s^−1^ within the same potential range.

The prevalence and character of the newly observed, quasi-reversible OER peak was further investigated in the 0.1 M TBAClO_4_/0.01 M Ca(ClO_4_)_2_/DMSO electrolyte at a range of slower scan rates (Fig. 3). Therein, CVs were collected at an Au electrode at scan rates from 1–100 mV s^−1^, after the completion of 150 cycles at 100 mV s^−1^ to allow the formation and stabilisation of the new OER peak. Therein, a clear OER peak is observed at similar potentials for scan rates from 100 to 5 mV s^−1^, while only a very small feature is observed at 2 mV s^−1^ and this is not present in the 1 mV s^−1^ scan (see inset in Fig. 3). Conversely, when measured at GC (Fig. S8†), the quasi-reversible OER peak exhibits the largest magnitude and, subsequently, the OER feature remains distinctly observable even at the lowest scan rates. For both Au and GC electrodes, all CV traces exhibit similar current decay profile after the OER peak, proceeding quite quickly to negligible currents. However, when the potential of the GC working electrode is swept at 10 mV s^−1^ or slower, a clear shoulder-like feature appears after the initial peak. As similar feature may be observed at Au with much less clarity due to the considerably lower current densities. This feature may indicate a multi-step process that is hidden within the peak in faster scans and, along with critical effects of the substrate materials, will be the subject of future investigations. Importantly, however, the quasi-reversible OER process at *ca.* −0.8 to −1 V, that is only observed to evolve with progressive cycling in this electrolyte formulation, remains present in some form at slow scan rates, especially for GC even when the electrode is swept as slow as 1 mV s^−1^.

Since the new OER feature arising in these formulations (*i.e.*Fig. 2(b)) shows a similar oxidation peak potential of the OER process compared to that in the Ca^2+^-free electrolyte (*i.e.*Fig. 1(a)), it might be suggested whether or not the occurrence of this oxidation peak is merely because of the depletion of Ca^2+^ at such a low concentration of 0.01 M. Therein, the TBA^+^-induced ORR/OER could gradually become predominant after all Ca^2+^ cations are consumed (by the formation and presumed precipitation of insoluble Ca-oxide products from reaction with reduced O_2_ species). Therefore, to probe this concept further, two experiments were conducted. For the first experiment, two clean Au electrodes were cycled through successive sets of 100 cycles in the same O_2_-saturated electrolyte (0.1 M TBAClO_4_/0.01 M Ca(ClO_4_)_2_/DMSO) (see Fig. S9† and corresponding discussion). Therein, immediately after 100 cycles, the first electrode was removed from solution and replaced with a second pristine Au electrode and a further 100 cycles were initiated immediately. If the Ca^2+^ was indeed completely consumed during the ORR at the first electrode, CVs on the second electrode would be expected to immediately resemble the final cycles at the first electrode. However, the two electrodes exhibit similar CV patterns, with the growth of the OER peak occurring only after successive cycling, indicating that the depletion of Ca^2+^ in the bulk electrolyte is not the reason for the OER peak in Fig. 2(b).

The second experiment using a rotating disc electrode (RDE) was utilised to isolate/exclude issues relating to depletion of the Ca^2+^ in solution local to the electrode surface. Therein, 50 CV cycles in O_2_-saturated 0.1 M TBAClO_4_/0.01 M Ca(ClO_4_)_2_/DMSO were measured at the GC RDE without rotation to allow for the evolution of the OER peak as seen in Fig. 2(b). Subsequently, the electrode was rotated for 30 min at 900 rpm with O_2_ bubbling, then stopped and 50 more CV cycles were collected. The resulting CVs are presented in Fig. S10 in the ESI.† Critically, even after rotation and, therefore, effective remixing and homogenisation of the bulk electrolyte, the quasi-reversible OER peak remains. If the OER peak evolved on cycling due to depletion of Ca^2+^ within the diffusion layer local to the electrode surface, breaking/resetting this diffusion layer by extensive rotation and gas bubbling would be expected to remove (or reduce) the presence of the OER peak. Consequently, it can be inferred the growth, and persistence, of the OER peak arises from species forming at or on the electrode surface.

**Fig. 4:**
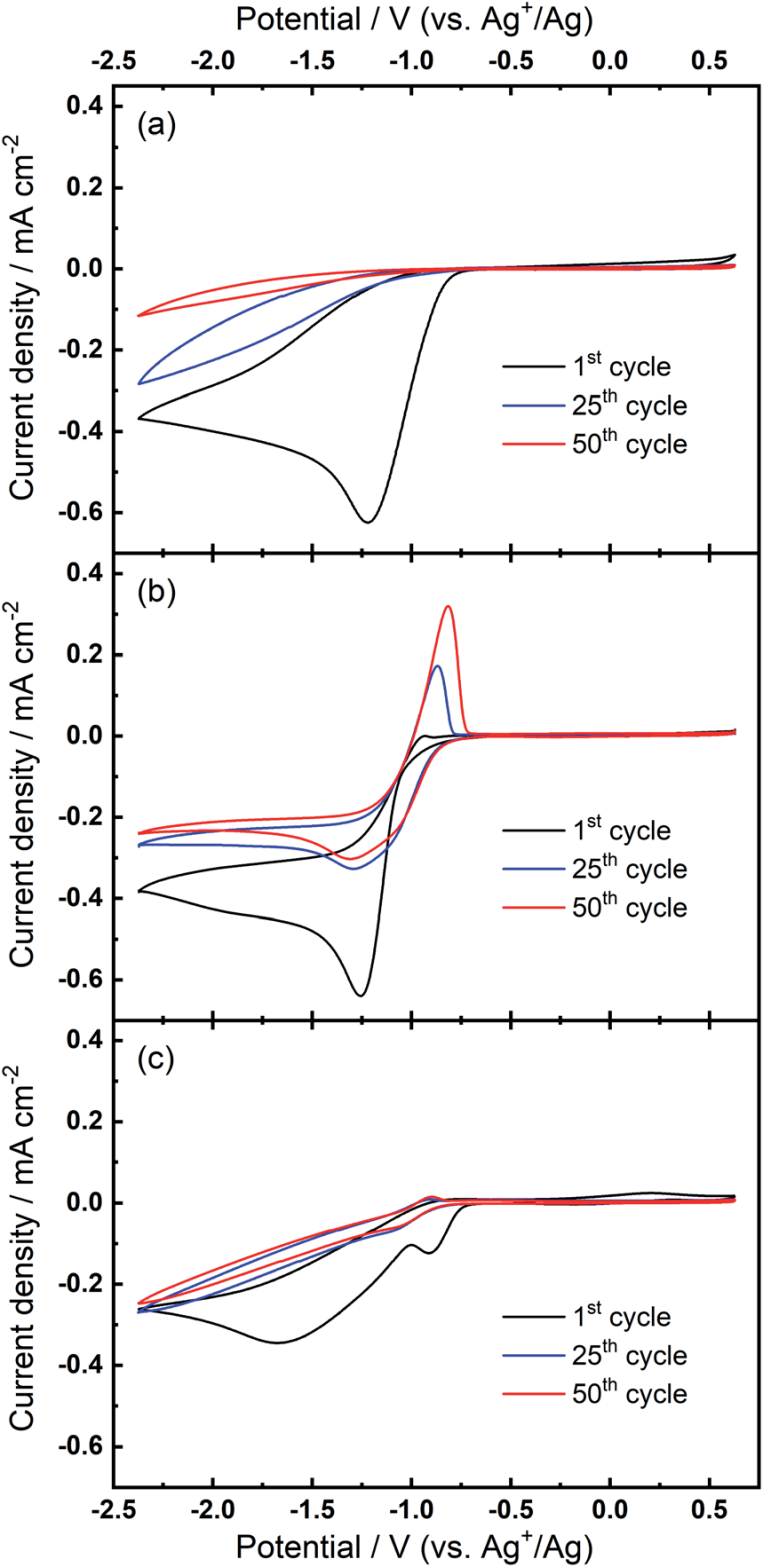
Comparative cyclic voltammograms of the ORR and OER on Au at 100 mV s^−1^ in O_2_-saturated electrolytes of (a) Electrolyte 1 (0.01 M Ca(ClO_4_)_2_/DMSO), (b) Electrolyte 2 (0.1 M TBAClO_4_/0.01 M Ca(ClO_4_)_2_/DMSO) and (c) Electrolyte 1 again. All CVs were measured using the same electrode.

Further investigations towards understanding the interplay of the TBA^+^/Ca^2+^ cations with the observed growth of the OER peak were undertaken. The OER peak is located at a similar potential where free O_2_^−^ is oxidised in the electrolyte containing only TBA^+^ (see Fig. 1(a)). However, the slow growth of this OER peak in Ca^2+^-containing electrolyte suggests some differences. Therefore, a series of comparative experiments were designed and carried out to further probe the effect of TBA^+^ and Ca^2+^ in this electrolyte. Two electrolytes were prepared in two individual cells. Electrolyte 1 contained 0.01 M Ca(ClO_4_)_2_ in DMSO, whilst Electrolyte 2 contained 0.1 M TBAClO_4_ and 0.01 M Ca(ClO_4_)_2_ in DMSO, with both electrolytes being saturated with O_2_. CV measurements would be performed at the same Au electrode in these two electrolytes in the order: Electrolyte 1, Electrolyte 2, and then Electrolyte 1 again, with a brief washing step of the working electrode in pure DMSO in between each step. Firstly, CV was recorded in the Electrolyte 1 for 50 cycles (Fig. 4(a)), wherein no OER peak could be found, consistent with Fig. 1(b). Then, the working electrode was transferred to Electrolyte 2 for the successive measurement (Fig. 4(b)), where parameters of the CV remained the same. In this electrolyte, the OER peak, which is observed in Fig. 2(b), appears and grows after successive cycling. Subsequently, the same working electrode was moved back to Electrolyte 1 and the same CV measurement was repeated (Fig. 4(c)). Therein, the OER peak seen in Fig. 4(b) (and Fig. 2(b)) disappears, suggesting that the occurrence of the reversible ORR/OER peaks is dependent on the presence of TBA^+^ within the electrolyte.

It is noted that the cycling behaviour in Fig. 4(c) appears somewhat different from that in Fig. 4(a) despite the electrolyte being the same for both steps (*i.e.*, Electrolyte 1). Firstly, the initial cycle in the repeated step in Electrolyte 1 (Fig. 4(c)) gives a larger current density than in the final cycle in first step (Fig. 4(a)). This is, in part, due to the repeated bubbling of the Electrolyte 1 with O_2_ prior to initiating the CVs and indicates the passivation effects observed in the first step are not permanent. Secondly, an additional peak at *ca.* −0.8 V in Fig. 4(c) is attributed to the residual reactants and/or ORR intermediates/products brought from the Electrolyte 2 and hence remaining at the electrode surface. Thirdly, the reductive current densities measured after 50 cycles are larger in the repeated step (Fig. 4(c)) than for when this electrolyte is first used (Fig. 4(a)) and the formation of a small OER peak can be seen at cycle 50. Therein, while bulk O_2_ concentrations may be different between the repeated measurements, the larger reduction currents are indicative of reduced passivation of the Au surface at high cycle numbers. Notably the passivation (*i.e.* suppression of the reduction current densities) is even less severe in the presence of TBA^+^ (Electrolyte 2). As such, the TBA^+^ has a clear effect on the nature of product formation, as well as the overall reversibility, in these formulations. Consequently, the electrochemically-generated and partially-passivating interlayer remaining at the Au surface (formed in Electrolyte 2) has an influence on the subsequent electrochemistry, even in the absence of the TBA^+^ salt in the bulk electrolyte. However, it is unclear whether the new very small OER-type peak originates from this interlayer, or simply any residual TBA^+^ components not removed during the washing step.

**Fig. 5:**
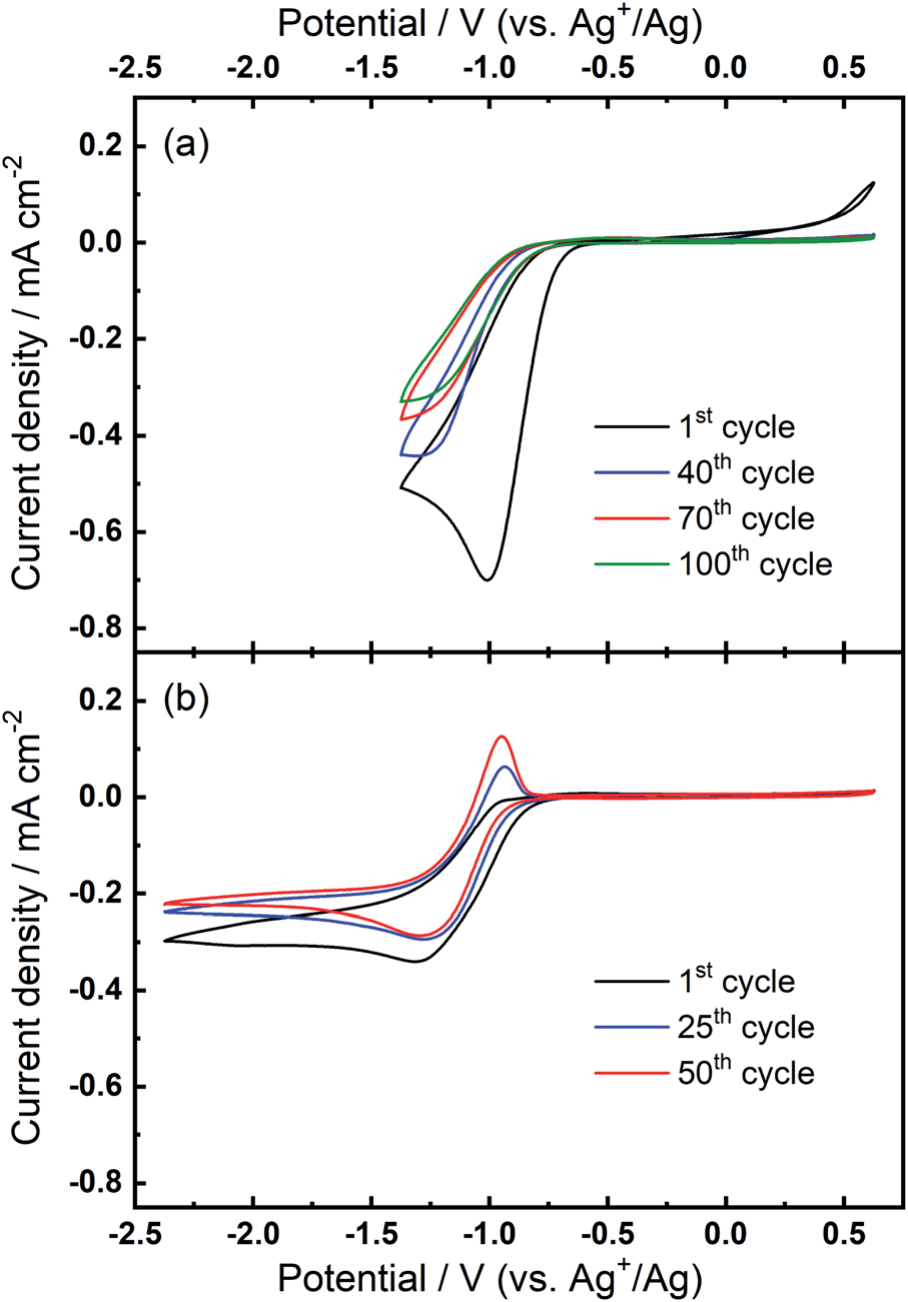
Comparative cyclic voltammograms of the ORR and OER on Au at 100 mV s^−1^ within (a) the reduced potential window (−1.37 V to 0.63 V) and, subsequently, (b) the expanded potential window (−2.37 V to 0.63 V) in O_2_-saturated 0.1 M TBAClO_4_/0.01 M Ca(ClO_4_)_2_/DMSO.

The prior CV experiments suggest that TBA^+^ plays a crucial role in these relatively reversible ORR/OER processes, because using Ca^2+^ alone cannot result in any OER peak. However, the origin of the growth of the OER peak in 0.1 M TBAClO_4_/0.01 M Ca(ClO_4_)_2_/DMSO requires further investigation. Note that the OER peak potential is similar to the oxidation peak of free superoxide (oxidation of O_2_^−^, see Fig. 1(a) and corresponding discussion) despite slight differences in the shape/position of the OER peak. Thus, we applied a smaller potential window (*i.e.* smaller reductive overpotential but the same oxidation overpotential), where the reversible O_2_^−^ formation/oxidation redox peaks are involved in the comparative case of 0.1 M TBAClO_4_/DMSO, to examine whether this OER peak appears. Surprisingly, Fig. 5(a) exhibits no OER peak growing over cycles within the smaller potential region where the O_2_^−^ formation could be expected to be favoured. Within the same cell, expansion of the negative potential range in subsequent CVs (Fig. 5(b)) leads to the occurrence and continuous growth of the OER peak, in agreement with Fig. 2(b). Accordingly, the occurrence of the oxidation behaviour is potential-dependent, where it is activated by sweeping to −2.37 V (*vs.* Ag^+^/Ag) and is not completely related to the free O_2_^−^ species formed in the electrolyte that only contains the TBAClO_4_ salt (see Fig. 1(a)).

Combining these observations, the relatively reversible ORR/OER with small overpotential for re-oxidation can be rationalised by the competition between the TBA^+^ and Ca^2+^ cations to interact with O_2_^−^. Firstly, Ca^2+^ is the prevailing cation when TBA^+^ and Ca^2+^ are at the same concentration (0.1 M), similar to the fact that adding a small amount of Li^+^ into TBA^+^-containing electrolyte can substantially alter the oxygen electrochemistry and reduce the reversibility.^6,16^ As Fig. 1(b) shows, the presence of Ca^2+^ results in irreversible ORR/OER, because the Ca^2+^-induced ORR process dominates and this explains why no oxidation peak of ORR species can be seen in Fig. 2(a). By contrast, when the concentration of TBA^+^ is much higher than Ca^2+^ (10 TBA^+^:1 Ca^2+^), Ca^2+^ can only prevail in a few early cycles and then TBA^+^ starts to partially participate in the oxygen reduction process and hence influence the electrochemistry, forming a pair of TBA^+^-induced ORR/OER redox peaks, as shown in Fig. 2(b). Secondly, Fig. 5(a) reveals that if the reductive overpotential is not sufficient, there is no OER peak similar to that in Fig. 5(b) even though TBA^+^ has a much higher concentration. This phenomenon can be explained by the fact that the ability of TBA^+^ to adsorb to the electrode surface is enhanced with the increasing overpotential, due to the conformational change of TBA^+^ cation.^20,34,35^ In other words, the TBA^+^ can more easily approach/adsorb to the electrode/electrolyte interface at larger reduction overpotentials. Overall, providing the TBA^+^ is more competitive (*i.e.* higher concentration and larger reductive overpotential), the characteristics of TBA^+^-induced ORR/OER appear. To verify this argument, another CV experiment with equivalent concentrations of both salts (0.1 M TBAClO_4_/0.1 M Ca(ClO_4_)_2_/DMSO) was performed in a more extended electrochemical window, as shown in Fig. S11.† Therein, unlike Fig. 2(a), the appearance of OER peak can be observed when cycled with larger reductive overpotentials (see Fig. S11† and corresponding discussion). As shown by the comparison of CVs in two different electrochemical windows, a larger reductive overpotential renders the TBA^+^ more competitive and more able to participate, to higher degrees, in the ORR/OER processes.

**Fig. 6:**
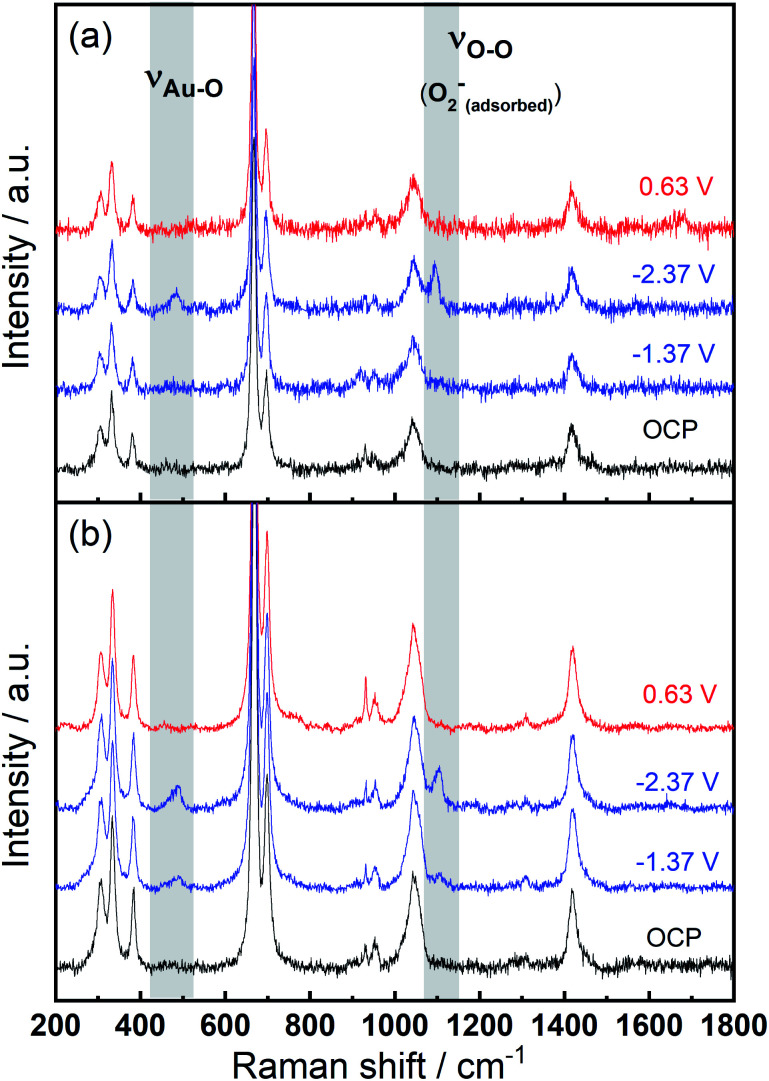
*In situ* SERS (normalised) measured at various potentials on a roughened Au electrode in O_2_-saturated (a) 0.1 M TBAClO_4_/0.01 M Ca(ClO_4_)_2_/DMSO and (b) 0.1 M TBAClO_4_/DMSO.

To identify speciation at the electrode surface under potential control, *in situ* SERS was conducted upon a roughened Au electrode. The intensity of the obtained spectra were normalised against the intense peak at 668 cm^−1^ that is assigned to the symmetric stretching mode of the C–S bond in DMSO.^36^Fig. 6(a) displays the Raman spectra in 0.1 M TBAClO_4_/0.01 M Ca(ClO_4_)_2_/DMSO. Firstly, a Raman spectrum is collected at the open circuit potential (OCP) prior to the electrochemical measurements. At OCP, all the observed peaks can be ascribed to the electrolyte (both DMSO and ClO_4_^−^ anion, see assignments in Table S1†). Then Raman spectrum is recorded at −1.37 V, where no discernible changes to the spectrum can be observed. When the potential is fixed at −2.37 V, the growth of two peaks can be detected at 486 cm^−1^ (*ν*_Au–O_) and 1099 cm^−1^ (*ν*_O–O_) and these are assigned to the adsorbed superoxide at the Au surface.^16,17,37,38^ These two Raman bands disappear as the electrode potential is fixed at an oxidative potential of 0.63 V. Fig. 6(a) is consistent with the electrochemical responses displayed in Fig. 5, where only a large reductive overpotentials (−2.37 V) can induce the TBA^+^-supported ORR behaviour, as indicated by the occurring OER peak. In Fig. 6(b) the electrolyte of 0.1 M TBAClO_4_ is tested for comparison and it shows two similar vibration bands of superoxide (490 and 1107 cm^−1^, respectively) as well. In the absence of Ca^2+^, free superoxide (also referred to as TBA^+^--O_2_^−^ throughout this work) can be generated and detected even though the reductive overpotential is not large (−1.37 V). By comparing the two bands in Ca^2+^-containing and Ca^2+^-free electrolytes, the similar band positions (486 and 1099 cm^−1^*vs.* 490 and 1107 cm^−1^) suggest a relatively close nature of superoxide species in Ca^2+^-containing/Ca^2+^-free electrolytes. It is expected that there would be a significant band shift when the superoxide is bound to a metal ion, *e.g.*, Ca(O_2_)_2_, taking into account the consistent examples of Li^+^- and Na^+^-containing DMSO electrolytes reported in the literature (1110/1132 cm^−1^ for O_2_^−^/LiO_2_,^6^ 1110/1138 cm^−1^ for O_2_^−^/LiO_2_,^39^ 1107/1156 cm^−1^ for O_2_^−^/NaO_2_,^37^ 1105/1158 for O_2_^−^/NaO_2_,^38^ respectively). Furthermore, the Raman spectrum of CaO_2_ (Fig. S12† and corresponding discussion) exhibits a peak at 840 cm^−1^, in agreement with the O–O^2−^ bond (842 cm^−1^) of BaO_2_ sample,^40^ and with the observed discharge product assignment in an intermetallic CaLi_2_–O_2_ battery discharged using a Ca^2+^-based electrolyte.^41^ However, no similar peak is observed in the *in situ* Raman spectra of the electrode/electrolyte interface presented in Fig. 6. Note that no other new peaks beyond those assigned to *ν*_Au–O_ and *ν*_O–O_ can be observed, and thus assigned to other ORR intermediates/products, in the *in situ* Raman spectra.

**Fig. 7:**
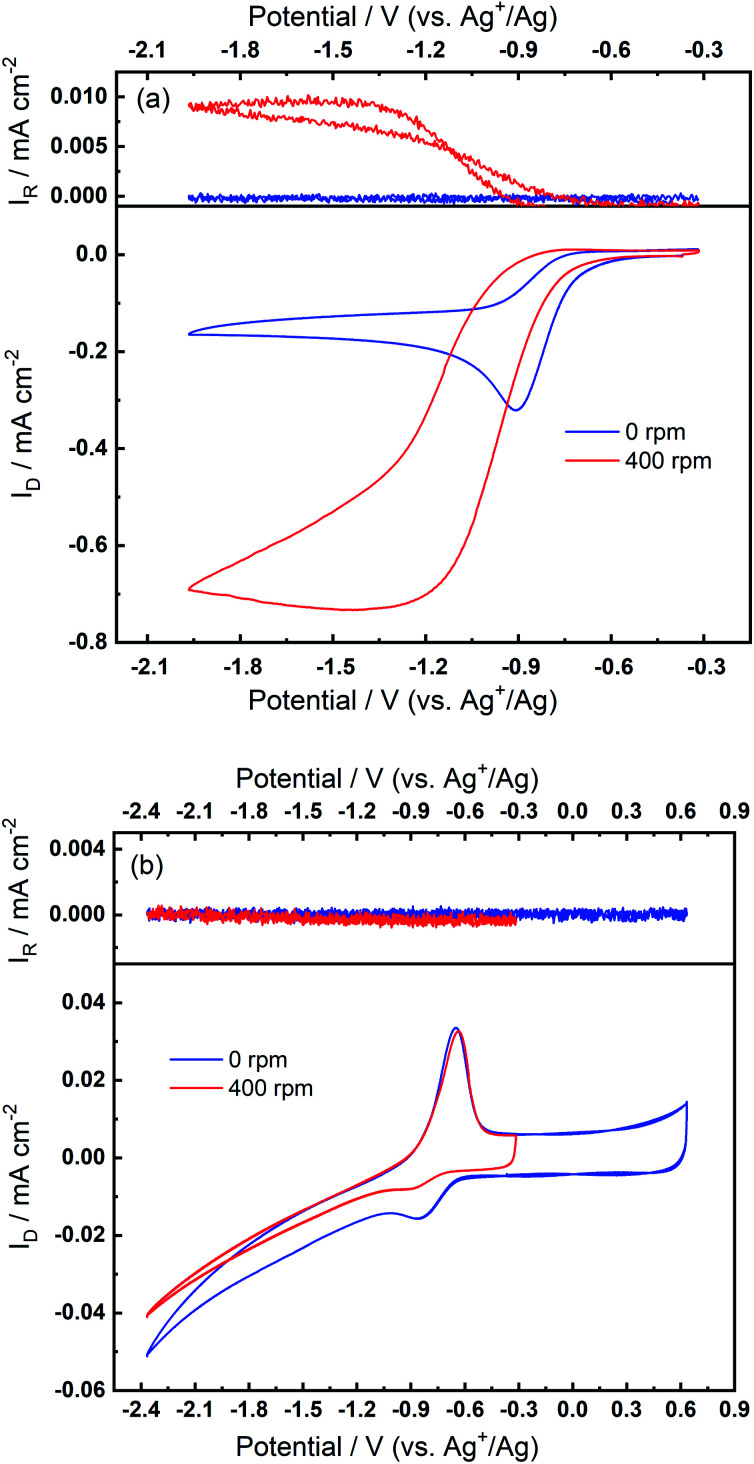
RRDE measurements at 100 mV s^−1^ with rotation speeds of 0 rpm and 400 rpm (a) before and (b) after the oxidation peak appears in O_2_-bubbled 0.1 M TBAClO_4_/0.01 M Ca(ClO_4_)_2_/DMSO. The disc and ring electrodes are GC and Pt, respectively, and the potential of ring is −0.37 V. A potential limit of −2.37 V is set in (b), in accordance with previous CVs wherein the reversible OER peak can be found.

The superoxide species produced in 0.1 M TBAClO_4_/0.01 M Ca(ClO_4_)_2_/DMSO after successive cycling behaves similarly to the free O_2_^−^ in Ca^2+^-free TBAClO_4_/DMSO, according to the obtained CVs and SERS results. On the other hand, investigations were performed on a RRDE (disc: GC, ring: Pt) to characterise the ORR process under a forced convection of electrolyte. If the free O_2_^−^ is generated in the ORR, the ring electrode (fixed at −0.37 V) should be able to capture this intermediate and oxidise it (see Fig. S13† and corresponding discussion). In Fig. 7(a), CVs on the disc and corresponding ring responses measured at 0 rpm and 400 rpm are presented. There is a large hysteresis loop on the negative sweep upon rotation, in accordance with our observation that the surface is being passivated over CV cycles, which leads to the deactivation of electrode.^24,42,43^ Thus, the reduction current does not follow the behaviour under pure mass transport control. With respect to current responses on the ring electrode, a limiting current is recorded. The ring current substantiates that some soluble superoxide species forms when the disc electrode is relatively clean and not passivated (*i.e.* experiencing less CV cycles and smaller reduction overpotentials). However, the amount of superoxide is relatively low (see Fig. S14† and corresponding discussion) and hence most of the ORR products are probably (per)oxide species or side reaction products.

After collecting data presented in Fig. 7(a), the electrode was scanned to −2.37 V for sufficient numbers of CV cycles without any rotation so that the oxidation peak could grow with time, and these CV scans were stopped after steady-state OER peaks were observed. After the OER peak appeared and stabilised, RRDE CVs were performed again. Fig. 7(b) shows that the disc current is invariant with the rotation speed and is much smaller (*ca.* 10 times) than the current in Fig. 7(a), which suggests again that a partial passivation layer forms on the disc, preventing the diffusion-controlled electrochemical response. On the disc electrode, an OER peak can be seen both when static and under rotation. Critically, when rotation is applied, no ring current is observed and the OER peak magnitude on the disc electrode is unaffected. The OER peak on the disc indicates the formation of species which can be re-oxidised after the generation of the partially passivating layer. Furthermore, the absence of a current response at the ring under rotation, and the persistence of the OER peak when static or rotated, indicates that this species is somewhat confined within the partial passivation layer (formed with continued electrochemical cycling) on the disc electrode and is hardly soluble in the bulk electrolyte. These observations are also consistent when a faster rotation speed (900 rpm) is applied to the system (see Fig. S15 in the ESI†).

**Scheme 1:**
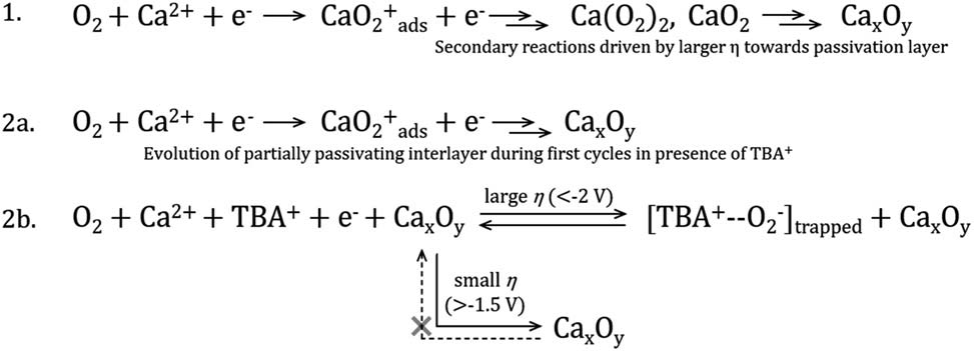
Proposed ORR mechanism in DMSO electrolytes containing either (1) only Ca^2+^ cations or (2) both TBA^+^ and Ca^2+^. Line 2a represents the first few cycles at the pristine Au electrode where no quasi-reversible OER is observed, whereas line 2b illustrates the new trapped interfacial redox of the [TBA^+^--O_2_^−^] species at the Ca_*x*_O_*y*_/Au interface under larger overpotentials (*η*). Voltages approximated from experimental work but specific critical limits will also depend on electrolyte composition and salt concentrations.

Based on the discussions thus far, a mechanism is proposed to elucidate the possible ORR process in the TBAClO_4_/Ca(ClO_4_)_2_/DMSO electrolyte (see Scheme 1). When the ORR onset potential is reached in the negative sweep, free superoxide (O_2_^−^) is first produced, because the ORR has similar onset potentials in Ca^2+^-containing and Ca^2+^-free electrolytes (also see O_2_/O_2_^−^ redox potential by Reinsberg and co-workers^23^). This generated superoxide species can be stabilised by interactions with either the TBA^+^ cation or the Ca^2+^ cation, which is dependent on the relative competitiveness of TBA^+^ and Ca^2+^. In general, Ca^2+^ is dominating and more favoured by the O_2_^−^, but weak interactions between TBA^+^ and O_2_^−^ (TBA^+^--O_2_^−^), that do not destabilise the superoxide and promote the formation of peroxide, can also form and then lead to a small OER peak as the TBA^+^ becomes more competitive (with larger reduction overpotentials and/or higher concentrations of TBA^+^). Ca^2+^-stabilised O_2_^−^, probably CaO_2_^+^ or Ca(O_2_)_2_, tends to be reduced, disproportionated or decomposed rapidly to a final product which cannot be re-oxidised in the investigated potential window, and this final product then (partially) passivates the electrode surface. However, reversible ORR/OER induced by TBA^+^ can still proceed to some degree on or within the passivation layer. *In situ* SERS confirms the presence of TBA^+^--O_2_^−^ at the electrode/electrolyte interface, while the RRDE shows that the superoxide is not soluble in the electrolyte, suggesting that the TBA^+^-interacting O_2_^−^ may be trapped in or strongly adsorbed on the passivation layer. It is noteworthy that Reinsberg *et al.* proposed a similar ORR mechanism in Mg^2+^-containing DMSO, but without spectroscopic confirmation.^23^ In comparison, in this work direct experimental evidence (*i.e.* relatively reversible ORR/OER redox couple) of the presence of O_2_^−^ intermediate in the first step of the ORR with the assistance of TBA^+^ cations by manipulating the salt concentrations and applied potential windows has been shown.

The observations and proposed ORR mechanism in the presence of both Ca^2+^ and TBA^+^ cations can be corroborated by Behm's work about the ORR/OER in Mg^2+^-containing ionic liquid 1-butyl-1-methylpyrrolidinium bis(trifluoromethylsulfonyl)imide ([BMP][TFSI]).^43,44^ They reported the appearance of a small anodic peak on Au and GC electrodes after a few CV cycles along with decreased ORR current over cycles.^44^ They also found that a more negative potential region results in a more pronounced OER peak. The observed OER peak is located at a similar potential to that of the OER peak observed in the Mg^2+^-free [BMP][TFSI]. The authors claimed that reversible ORR/OER can still occur, although significantly hindered, on the passivated electrode, which is consistent with our results. Considering their paper, we expect that the BMP^+^ cation of the ionic liquid, which is a similar bulky inert alkylammonium cation, acts similarly to the TBA^+^ cation. The much higher concentration of BMP^+^ (*i.e.* more competitive BMP^+^) than that used in our work renders a much faster appearance of the OER peak, after merely two CV cycles. These findings in this paper can be strong and direct supports for our observations, because there are no effects from solvents that need to be concerned and therefore, the whole system within ionic liquids can be less complex. Further work again published by Behm's group investigated the ORR/OER in the same ionic liquid, where RRDE was used.^43^ No ring signal can be seen while the quasi-reversible ORR/OER peaks are seen on the disc, and the authors suggested this is because the formed superoxide is adsorbed on or trapped within the electrochemically formed passivation layer. Both these papers are in good agreement with observation of the appearance of quasi-reversible OER peak and its characteristics demonstrated by both CV and RRDE measurements. Comparative experimental observations between Mg^2+^ and Ca^2+^ systems are particularly interesting given that the magnesium cation is smaller (86 *vs.* 114 pm), making Mg^2+^ a much harder acid. Furthermore, it is noted that the ring structure of the BMP^+^ cation used in the Mg^2+^ studies results in a different steric hindrance compared to the TBA^+^ cation used here, leading to varying stabilisation interactions with ORR products. However, both bulky tetralkylammonium cations are comparatively softer acids than the corresponding Mg^2+^/Ca^2+^ cation.

**Fig. 8:**
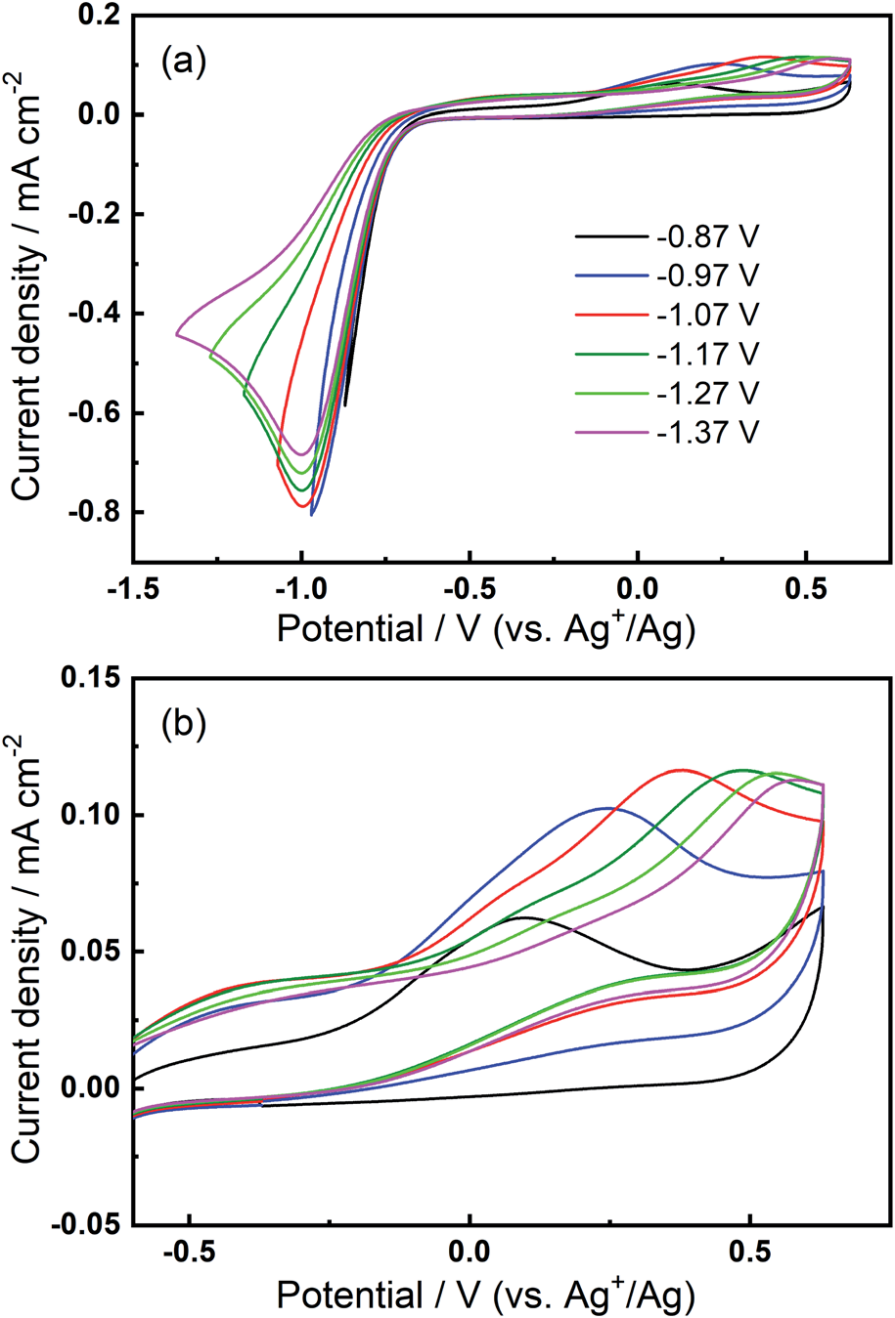
(a) Cyclic voltammograms on Au and (b) the magnified OER region at 100 mV s^−1^ within a reduced potential window (lower limit expanding from −0.87 V to −1.37 V) in O_2_-saturated 0.1 M TBAClO_4_/0.01 M Ca(ClO_4_)_2_/DMSO.

We then consider the reason that the effect of TBA^+^ and corresponding ORR/OER redox couple can be observed during the expansion of potential window (*i.e.* the lower potential limit expanding from −1.37 V to −2.37 V). Therein, an opposite change in potential window (*i.e.* lower potential limit >−1.37 V) is also investigated. An even smaller electrochemical window is investigated where the lower limit ranges between −0.87 and −1.37 V, as shown in Fig. 8. The first CV is swept to −0.87 V, and the reduction overpotential is gradually increased for each successive cycle to −0.97 V, −1.07 V, −1.17 V, −1.27 V, and ultimately −1.37 V, respectively. Fig. 8(a) shows no OER peak in the superoxide region (−0.9 to −0.7 V, refer to Fig. 2(b)) is observed, consistent with the phenomenon that TBA^+^ has little effect on the ORR with small reduction overpotentials, as shown in Fig. 5. Instead, a small OER peak can be obtained at more positive potentials greater than −0.2 V, and Fig. 8(b) shows the magnified OER peaks for clearer comparison. These more positive potentials than that for oxidation of free O_2_^−^ suggest a different species forms during the ORR when the reduction overpotential is small. Referring to the work published by Reinsberg and co-workers,^14^ the OER peak in a similar potential range (between 0 V and 0.5 V) is suggested to be the OER for contact ion pairs of Ca^2+^ and O_2_^−^. Also, considering the relatively large overpotential required for re-oxidation, this species is likely to be the CaO_2_^+^ or Ca(O_2_)_2_ intermediates in our proposed mechanism. A strong effect can be exerted by the Ca^2+^ cation on the O_2_^−^ anion, which results in a more stabilised species than TBA^+^--O_2_^−^. Thus, larger overpotentials would be required to oxidise Ca^2+^-stabilised O_2_^−^ species. It can be noted in Fig. 8(b) that, when the lower potential limit is shifted more negative, the corresponding OER peak shifts positively, indicating higher overpotential barriers for re-oxidation. This is likely to be the result of the accumulation of ORR intermediates (and/or decomposition products) that form the passivation layer on the electrode surface, leading to an increase in the OER overpotential. Also, the negative overpotential may accelerate the Ca^2+^-involved pathway in our mechanism, leading to formation of CaO_2_, CaO or even decomposition products which cannot be observed in the applied potential window. Fig. 8 supports the proposed mechanism by confirming the formation of Ca^2+^-stabilised superoxide species with lower reduction overpotentials. On the other hand, Fig. 8 also suggests that the presence of only minor anodic features in the region of *ca.* 0 V to 0.5 V in the Fig. S1 and S2† can be due to the larger negative potential window applied in these particular experiments. As long as the reduction overpotential is reduced, an anodic peak can become more prominent on the positive scan, in agreement with the literature.^14^ Overall, it can be concluded that the characteristic anodic peaks relating to the oxidation of CaO_2_^+^ (or Ca(O_2_)_2_) are easier to observe with smaller reduction overpotentials. Therein, the CaO_2_^+^ intermediate can last longer without the additional overpotential-driven secondary processes and, meanwhile, any effect of the TBA^+^ cation within this potential region is minimal or very short-lived. In contrast, if the reduction potential is sufficiently negative, the Ca^2+^-induced ORR pathway can proceed very fast to form CaO or other decomposition products that are hardly observable. Therein, the TBA^+^ becomes more competitive and begins to participate in the ORR, so the characteristic OER involving TBA^+^--O_2_^−^ can be seen.

Evaluating these observations of compositional and overpotential dependence on the emergence of the quasi-reversible TBA^+^--O_2_^−^ OER electrochemistry with progressive cycling in Ca^2+^-containing electrolytes, a mechanism of (partial) charge storage at this developing interface is considered; trapped interfacial redox. Therein, in the presence of Ca^2+^ (and TBA^+^), O_2_ reduction at pristine Au (as well as Pt and GC) yields CVs without large hysteresis on the reverse sweep (*i.e.*, no rapid passivation) and no evidence for quasi-reversible OER (in the −0.9 V to −0.7 V region). With progressive cycling to large overpotentials, ORR current peak magnitudes decay to broader plateaus but do not tend towards zero (over 100 cycles) and hysteresis remains minimal, indicating only partial passivation of the electrode by products of the ORR. Concurrently, the low-overpotential OER anodic peak (−0.9 V to −0.7 V), associated with the oxidation of free- or TBA^+^-interacting O_2_^−^ (confirmed by SERS), develops and grows with more cycling. Crucially, this does not occur in solutions containing only the Ca^2+^ salt and the excess concentrations of the alkylammonium cation are critical. The growth of this feature is exacerbated by larger reduction overpotentials on negative CV sweeps that drive the evolution of Ca_*x*_O_*y*_ product formation (Scheme 1) and the adsorption of the bulky TBA^+^ (in TBA^+^--O_2_^−^ pairings). While free or TBA^+^-interacting O_2_^−^ is a conventionally soluble species, the TBA^+^--O_2_^−^ generated at the new Ca_*x*_O_*y*_/Au surface is trapped or confined within the product matrix close to the electroactive Au surface (confirmed by RRDE voltammetry). Due to the reasonably large reductive overpotentials used here, as well as the expected reactivity of reduced oxygen, metal oxides and intermediates, the effect of decomposition reactions of electrolyte components on the formation of this partially passivating interlayer and the resulting new OER process cannot be ruled out at this stage. Indeed, prior work discussed earlier conducted on the O_2_ electrochemistry in the presence of Mg^2+^ (in [BMP][TFSI] ionic liquid) indicated the presence of additional inorganic and organic phases formed on the working electrode surface.^43,44^ Therein, this observation was classified by the authors as an electrode–electrolyte interphase originating from side reactions involving electrolyte components. Consequently, characterisation of the surface films formed here responsible for promoting the evolution of the new OER phenomenon will be an important area for future investigation.

Furthermore, it is also noteworthy that the quasi-reversible OER peak that arises in these electrolytes (*e.g.*, 100^th^ cycle in Fig. 2(b)) also arises at Pt and GC electrode substrates (and when the smaller TEA^+^-based salt is used), persists even at slow scan rates, and displays a non-Cottrellian decay of the current density succeeding the anodic peak. This is distinctly different from OER observed in the presence of only TBA^+^, directly compared in Fig. S5,† wherein measured currents depend on diffusion of soluble superoxide anions to the oxidising electrode surface. Collectively, these observations indicate a strong divergence from diffusion-control for this process in the presence of Ca^2+^, further supporting an apparent confinement of the species undergoing the observed OER at the electrode surface. Consequently, the O_2_/TBA^+^ OER evolves towards this *trapped interfacial redox* mechanism in the presence of Ca^2+^, facilitating the quasi-reversible, low-overpotential oxidation process that could have interesting implications on the rechargeability and efficiency of Ca–O_2_ batteries, and as a distinctive mechanism to store charge in general.

## 4. Conclusions

ORR/OER electrochemistry is reported in DMSO-based electrolytes containing both Ca^2+^ and TBA^+^ cations. A quasi-reversible (low overpotential) OER peak was found to appear and grow during multiple CV sweeps where TBA^+^ concentrations exceed that of the more reactive Ca^2+^ cation. The OER phenomenon was found to occur independently of the electrode substrate (Au, Pt or GC). A series of electrochemical tests confirmed that the OER peak is related to the presence of TBA^+^, the relative concentration of TBA^+^, and the applied potentials. The process can be described as resulting from competition between TBA^+^ and Ca^2+^ cations to interact with electrochemically generated O_2_^−^. *In situ* electrochemical SERS substantiated the formation of TBA^+^--O_2_^−^ and showed potential-dependent presence of O_2_^−^. Vibrational bands relating to either calcium superoxide or peroxide were not observed. RRDE showed that O_2_^−^, formed at the electrode interface in the presence of Ca^2+^, did not go into solution and the quasi-reversible OER remained confined at the electrode surface even under slower scan rates. This was ascribed to the adsorption or entrapment of O_2_^−^ (as TBA^+^--O_2_^−^) within a calcium oxide (Ca_*x*_O_*y*_) electrode interlayer. On the other hand, where conditions are optimised so that Ca^2+^-supported ORR pathway becomes slower, a larger overpotential OER process, distinct from the re-oxidation of TBA^+^--O_2_^−^ can be observed, supporting that two different ORR pathways caused by the TBA^+^ and Ca^2+^ cations are present. This study not only shows that both ORR and OER processes can occur in electrolytes that contain Ca^2+^ and provides a basis for further electrolyte development to enable the realisation of Ca–O_2_ batteries, but also introduces a mechanism of charge storage by trapped interfacial redox.

## Data availability

Data for this paper, including electrochemical and spectroscopic data, are available at https://doi.org/10.17638/datacat.liverpool.ac.uk/1309.

## Author contributions

Y-TL: majority of experimental work, writing original draft manuscript, review and editing, and visualisation. AN: experimental work and supervision, writing manuscript review and editing, and visualisation. C-CH: supervision, writing review and editing. LH: funding acquisition, project administration, supervision, writing manuscript review and editing.

## Conflicts of interest

There are no conflicts to declare.

## Supplementary Material

SC-012-D0SC06991D-s001
