# Supplementary material for: Trapped interfacial redox introduces reversibility in the oxygen reduction reaction in a non-aqueous Ca2+ electrolyte
Source: Chem Sci. 2021 May 28;12(25):8909–19. doi: 10.1039/d0sc06991d (PMC8246276; doi:10.1039/d0sc06991d)
Supplement: SC-012-D0SC06991D-s001 [file SC-012-D0SC06991D-s001.pdf]

Electronic Supplementary Material (ESI) for Chemical Science.
This journal is © The Royal Society of Chemistry 2021

Supporting Information

Trapped interfacial redox introduces reversibility in the oxygen reduction reaction in a non-aqueous Ca^{2+} electrolyte

Yi-Ting Lu, Alex R. Neale, Chi-Chang Hu*, Laurence J. Hardwick*

*Corresponding Authors: Laurence J. Hardwick, Professor
Stephenson Institute for Renewable Energy, Department of Chemistry,
University of Liverpool,
Liverpool L69 7ZD, UK

*Corresponding Authors: Chi-Chang Hu, NTHU Chair Professor
Department of Chemical Engineering,
National Tsing Hua University,
Hsin-Chu 300044, Taiwan

This supporting information includes **15 Figures** and **1 Table**.

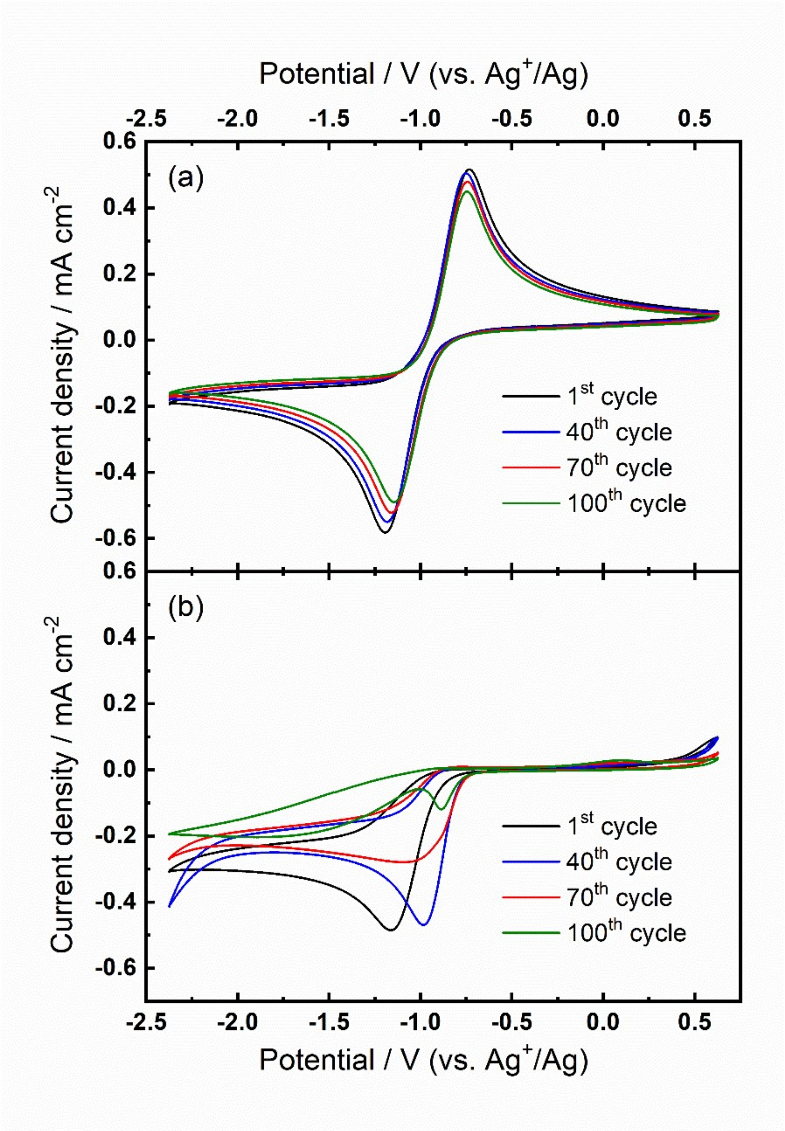

Figure S1. (a) Cyclic voltammograms of the ORR and OER at 100 mV s^{-1} on a Pt electrode in (a) 0.1 M $TBAClO_4$/DMSO and (b) 0.1 M $Ca(ClO_4)_2$/DMSO.

The reduction peak shifts over successive cycles in a similar trend to that on an Au electrode (**Figure 1(b)**). The reduction peak shifts positively in early cycles and shift negatively in late cycles. On the other hand, the Pt electrode shows an additional reduction peak at -0.9 V even at the 100th cycle, which is different from the flat reduction CV pattern for Au (**Figure 1(b)**) and GC (**Figure S2(b)**).

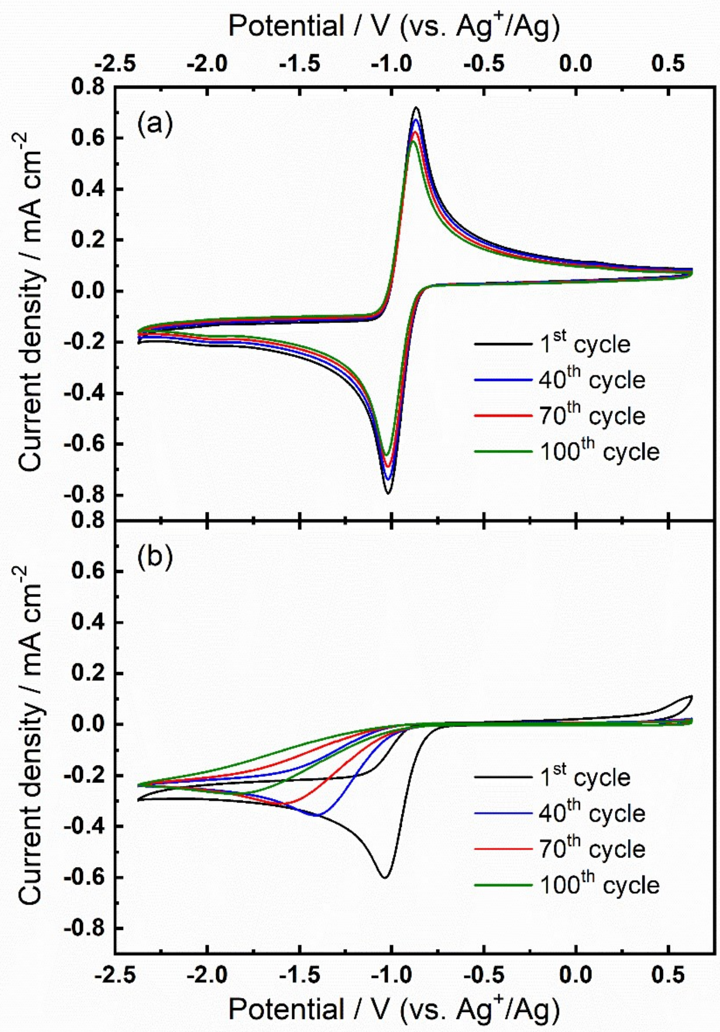

Figure S2. (a) Cyclic voltammograms of the ORR and OER at 100 mV s^{-1} on a GC electrode in (a) 0.1 M $TBAClO_4$/DMSO and (b) 0.1 M $Ca(ClO_4)_2$/DMSO.

The reduction peak shifts in a different way from those on Au (**Figure 1(b)**) and Pt electrodes (**Figure S1(b)**). Throughout the CV measurement, the reduction peak consistently shifts to negative potentials.

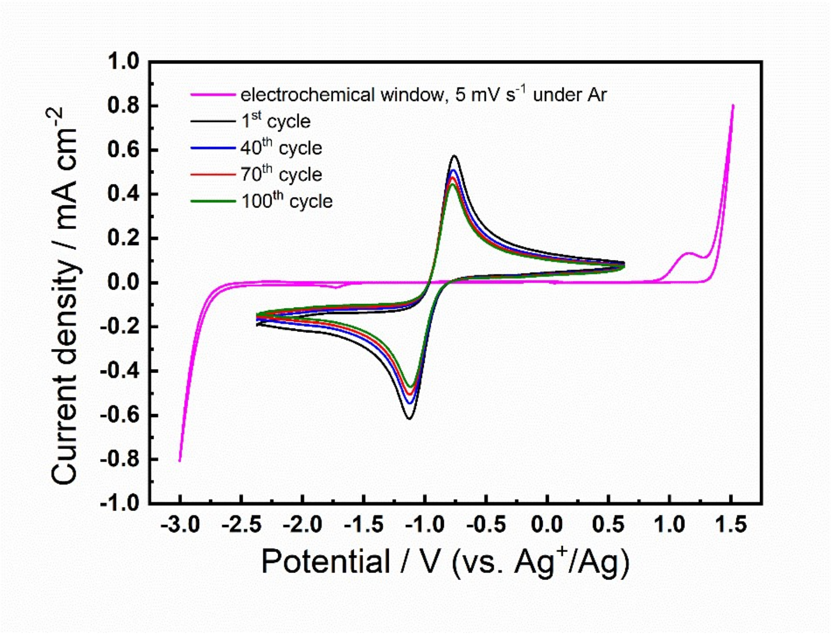

Figure S3. Electrochemical window, measured on a Au electrode at 5 mV s^{-1} in Ar-saturated 0.1 M $TBAClO_4$/DMSO. ORR/OER CVs measured on a Au electrode in the equivalent O_2-saturated electrolyte, taken from **Figure 1(a),** are overlaid for comparison.

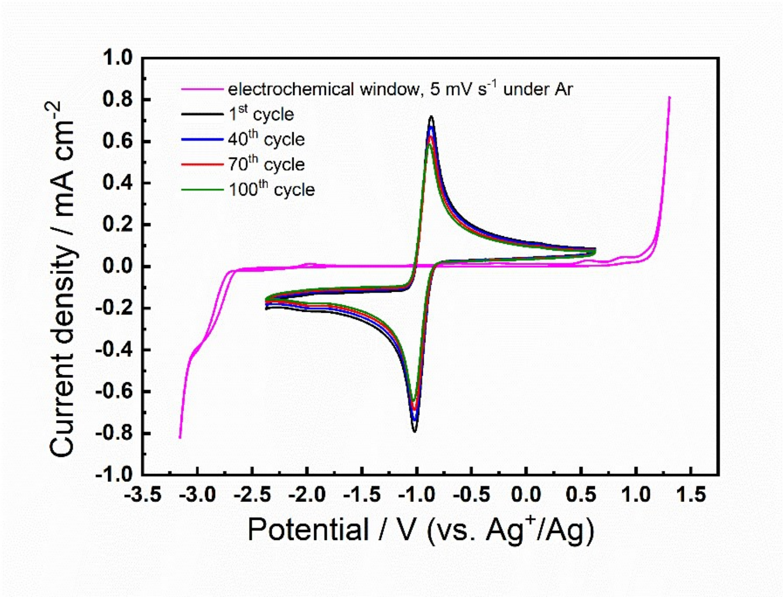

Figure S4. Electrochemical window, measured on a GC electrode at 5 mV s^{-1} in Ar-saturated 0.1 M $TBAClO_4$/DMSO. ORR/OER CVs measured on a GC electrode in the equivalent O_2-saturated electrolyte, taken from **Figure S2(a),** are overlaid for comparison.

The onset of reductive decomposition of bulk electrolyte at both electrodes (Figures S3 and S4) is estimated at -2.7 to -2.8 V using both a current density cut-off point of -0.1 mA cm^{-2} and the linear extrapolation to the *x*-axis of the massive electrolyte reduction current <-2.8 V.

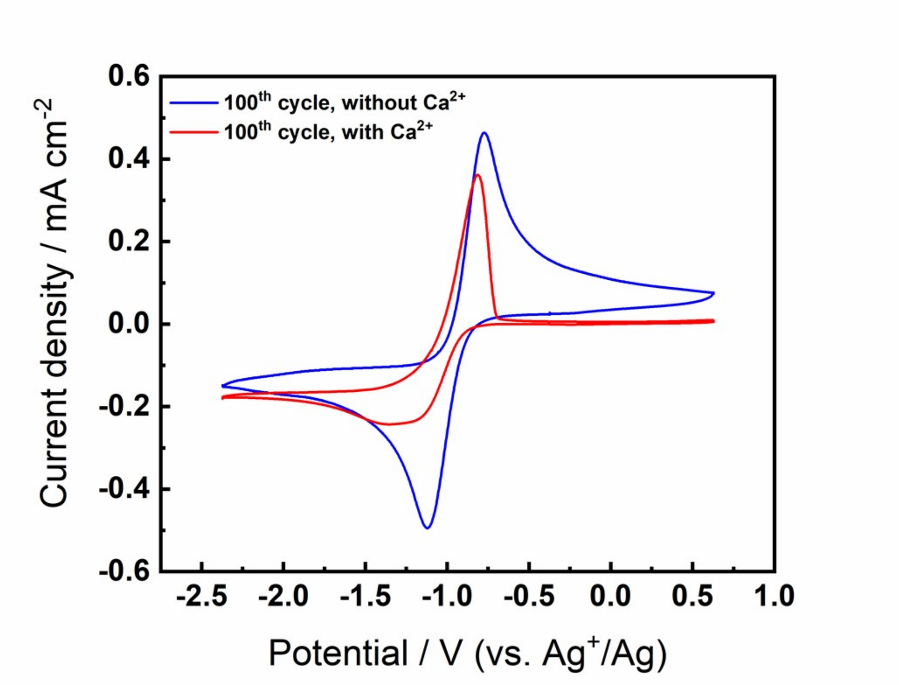

Figure S5. Cyclic voltammograms of the ORR/OER on Au after 100 cycles at 100 mV s^{-1} in O_2-saturated (blue) 0.1 M $TBAClO_4$/DMSO and (red) 0.1 M $TBAClO_4$/0.01 M $Ca(ClO_4)_2$/DMSO.

The comparative CVs taken after 100 cycles in O_2-saturated electrolyte are overlaid to highlight the characteristic difference in the profiles of the OER peaks. In the absence of Ca^{2+} (blue trace), a conventional Cottrellian current decay profile succeeds the peak current of the OER process dominated by diffusion of superoxide anions to the Au surface. Despite a drop in magnitude of the ORR/OER peaks, the profile of ORR/OER current response remains unchanged after 100 cycles in this electrolyte. In the presence of Ca^{2+} and TBA^+ after 100 cycles (red trace), the current profile after the OER peak decays rapidly, deviating away from conventional Cottrellian-type behaviour. In agreement with other observations discussed in the main text, this suggests a deviation away from a diffusion-controlled oxidation process as a result of changes at the Au surface after 100 ORR/OER cycles.

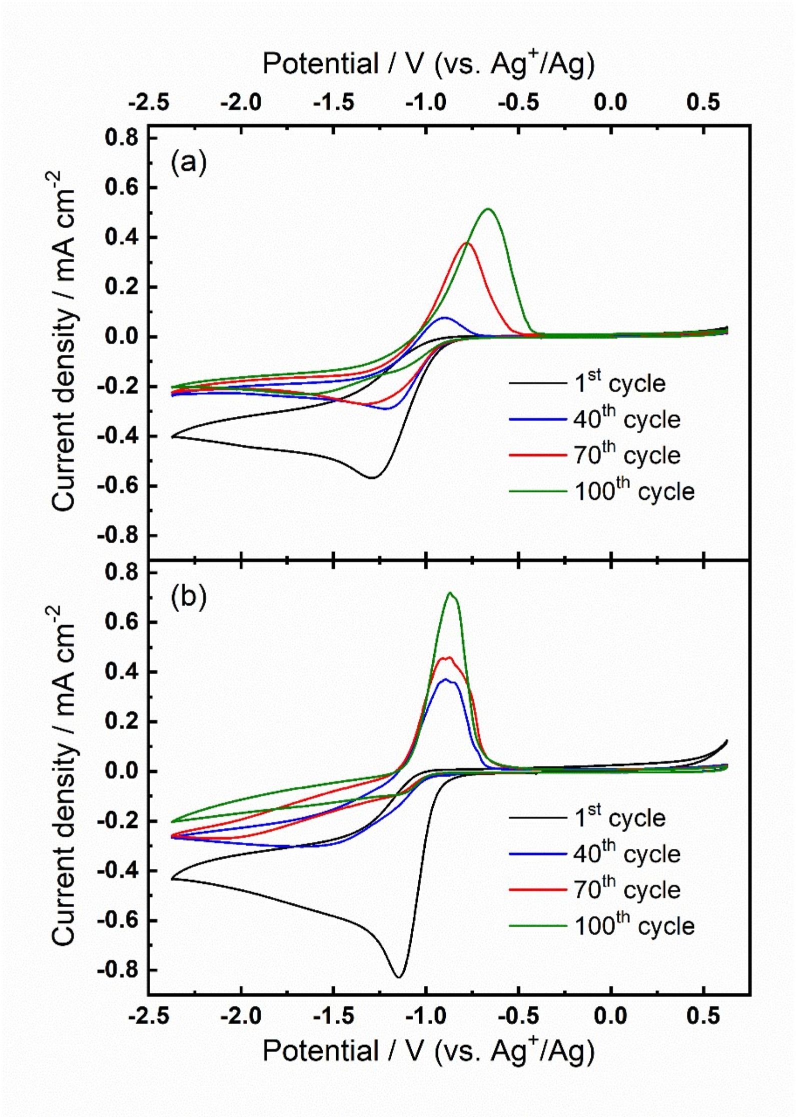

Figure S6. Cyclic voltammograms of the ORR and OER on (a) Pt and (b) GC electrodes at 100 mV s^{-1} in O_2-saturated 0.1 M $TBAClO_4$/0.01 M $Ca(ClO_4)_2$/DMSO.

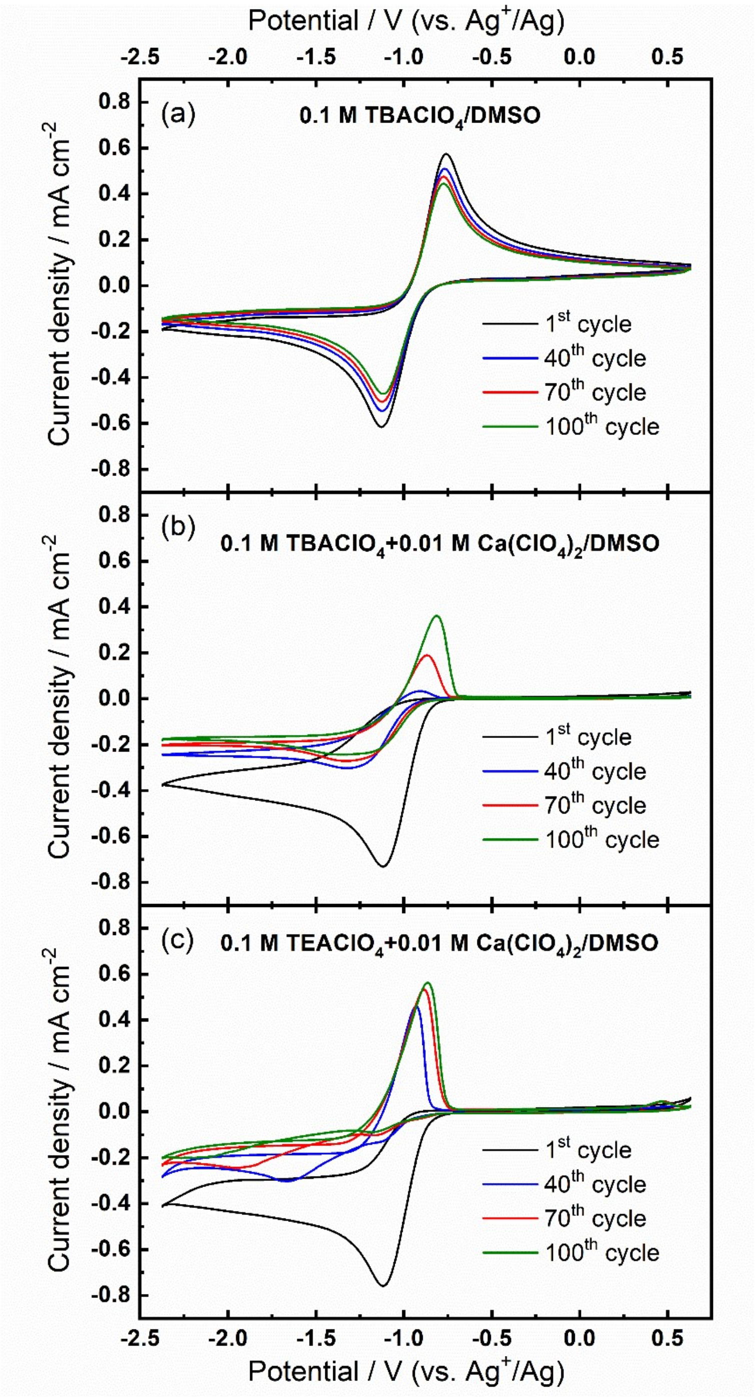

Figure S7. Cyclic voltammograms of the ORR and OER on Au electrodes at 100 mV s^{-1} for different O_2-saturated electrolytes.

Panel (c) shows the evolution of the new quasi-reversible OER peak reported in the TBA^+/Ca^{2+} electrolyte (b) is not exclusive to this formulation and is also observed when TBA^+ is substituted with the smaller analogous tetraethylammonium (TEA^+) cation.

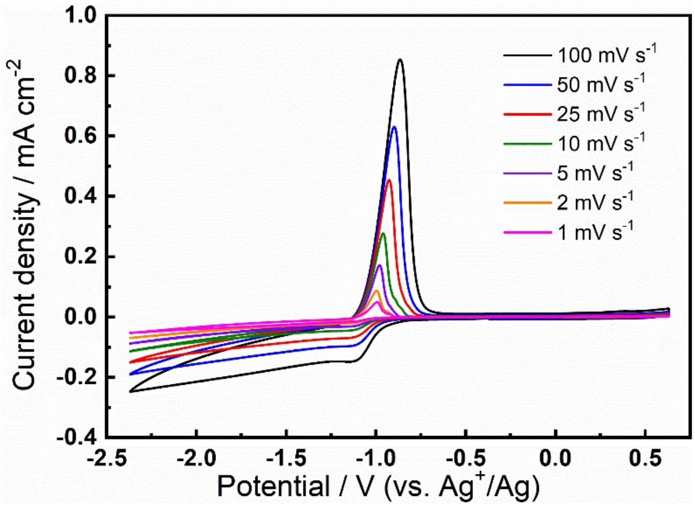

Figure S8. Cyclic voltammograms of the ORR and OER on GC at various scan rates in O_2-saturated 0.1 M $TBAClO_4$/0.01 M $Ca(ClO_4)_2$/DMSO. Variable scan rate voltammograms were collected after 100 cycles at 100 mV s^{-1} within the same potential range.

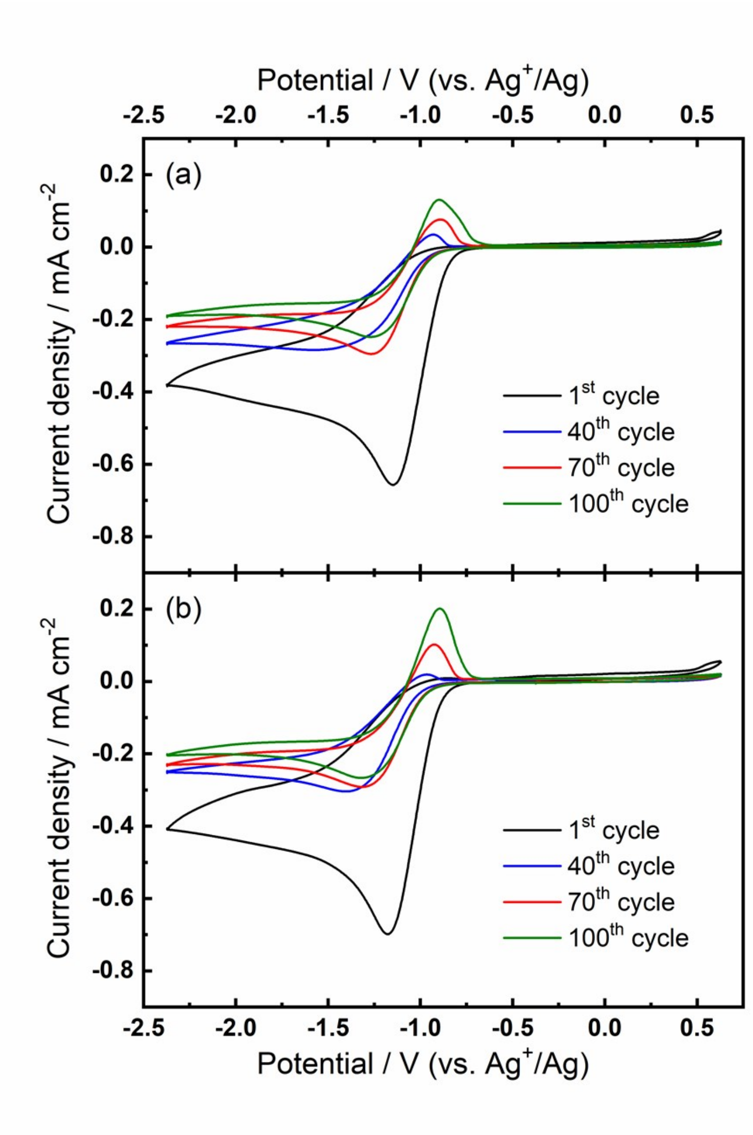

Figure S9. Two consecutive CV measurements at 100 mV s^{-1} on (a) the first Au electrode and (b) the second Au electrode in the same 0.1 M $TBAClO_4$/0.01 M $Ca(ClO_4)_2$/DMSO electrolyte.

Note that the CV patterns look almost the same although the OER peak intensities are slightly different. This indicates the Ca^{2+} bulk concentration is not reduced too seriously; otherwise the electrochemical behaviour should be changed significantly. Note that the electrolyte used was the same but was bubbled with oxygen for 30 min again before the second clean Au was set up and tested in the cell.

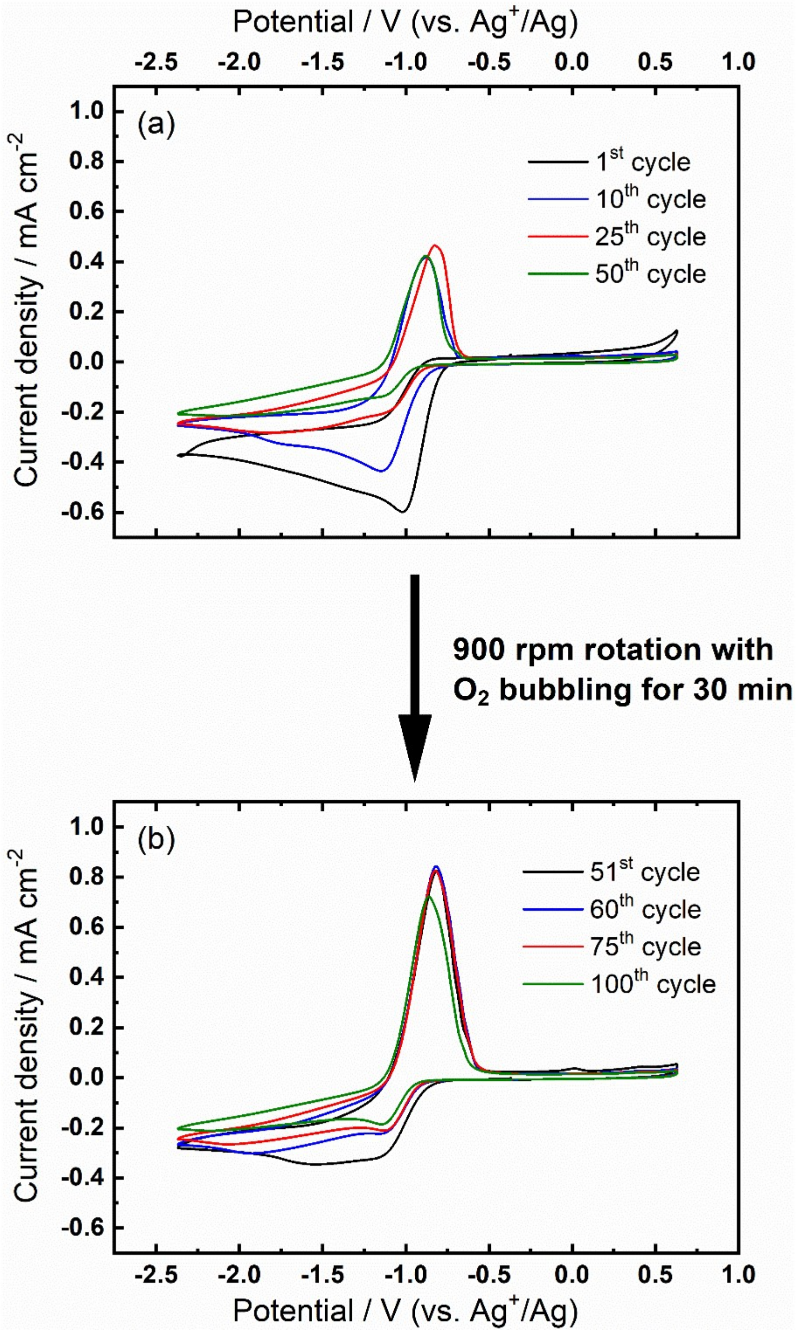

Figure S10. Cyclic voltammograms of the ORR and OER on GC RDE at 100 mV s^{-1} in O_2-saturated 0.1 M $TBAClO_4$/0.01 M $Ca(ClO_4)_2$/DMSO electrolyte. The electrode was first cycled for 50 cycles (a). The electrode was then held at OCV and rotated at 900 rpm for 30 min with bubbling of O_2. Subsequently, rotation and bubbling were halted and the electrode was cycled for a further 50 cycles (b).

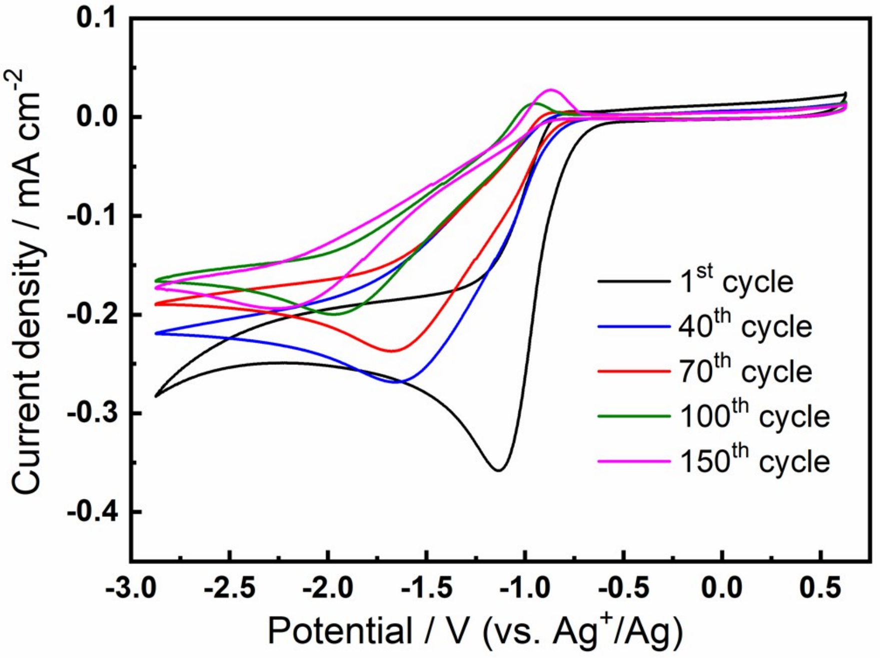

Figure S11. Cyclic voltammograms of the ORR and OER at 100 mV s^{-1} on a Au electrode in 0.1 M $TBAClO_4$/0.1 M $Ca(ClO_4)_2$/DMSO. A larger potential window (-2.87 V to 0.63 V) is used compare to that presented in **Figure 2(a)** in the main text (-2.37 V to 0.63 V).

To validate our statement that the appearance of the OER peak is due to the more competitive TBA^+ in **Figure 2(b)** and **Figure 5(b)**, we conducted the CV in 0.1 M $TBAClO_4$/0.1 M $Ca(ClO_4)_2$/DMSO, which does not show any OER peak in **Figure 2(a)**. An extra reductive overpotential (by 0.5 V) was applied and a growing OER peak can be found here, suggesting that TBA^+ can be involved in the ORR, to some extent.

Note that in this CV measurement, the OER peak is not obvious at the 100th cycle (green line). Thus, an extra 50 cycles were conducted to confirm the emergence of the quasi-reversible oxidation peak. The OER peak grows continually during these 50 cycles, which is a similar phenomenon to **Figure 2(b)**, **Figure 4(b)** and **Figure 5(b)**.

Table S1. Raman peak positions (cm^{-1}) and assignments at OCP for SERS measurements.

Peak position (cm^{-1})	Band Assignment
306	C-S-C out-of-plane bending
332	C-S-O out-of-plane bending
381	C-S-O in-plane rocking
668	C-S symmetric stretching
697	C-S asymmetric stretching
930	Cl-O stretching
952	H-C-H rocking
1041	S-O asymmetric stretching
1416	H-C-H deformation

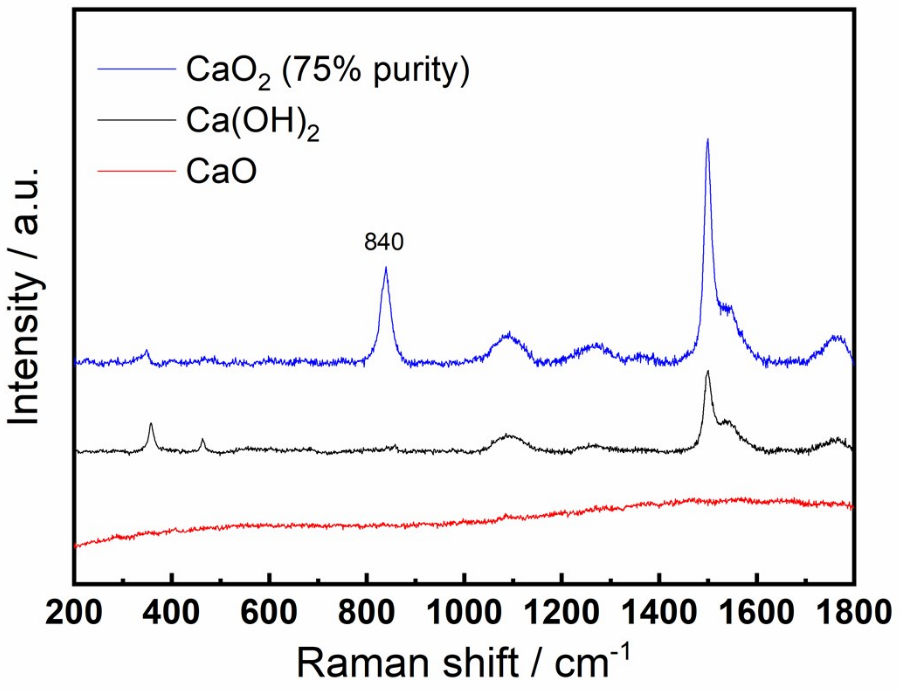

Figure S12. Raman spectra for commercial CaO_2 (75% purity), $Ca(OH)_2$ and CaO powders.

In **Figure S12**, CaO shows a featureless spectrum, while CaO_2 exhibits multiple peaks. However, because of the low purity from the commercial CaO_2 sample, most peaks are attributed to impurities such as $Ca(OH)_2$. The characteristic peak of CaO_2 is centred at 840 cm^{-1}, which agrees with the literature value (842 cm^{-1}) for BaO_2 (S. C. Su and A. T. Bell, *Catalysis Letters*, 1996, **36**, 15-19).

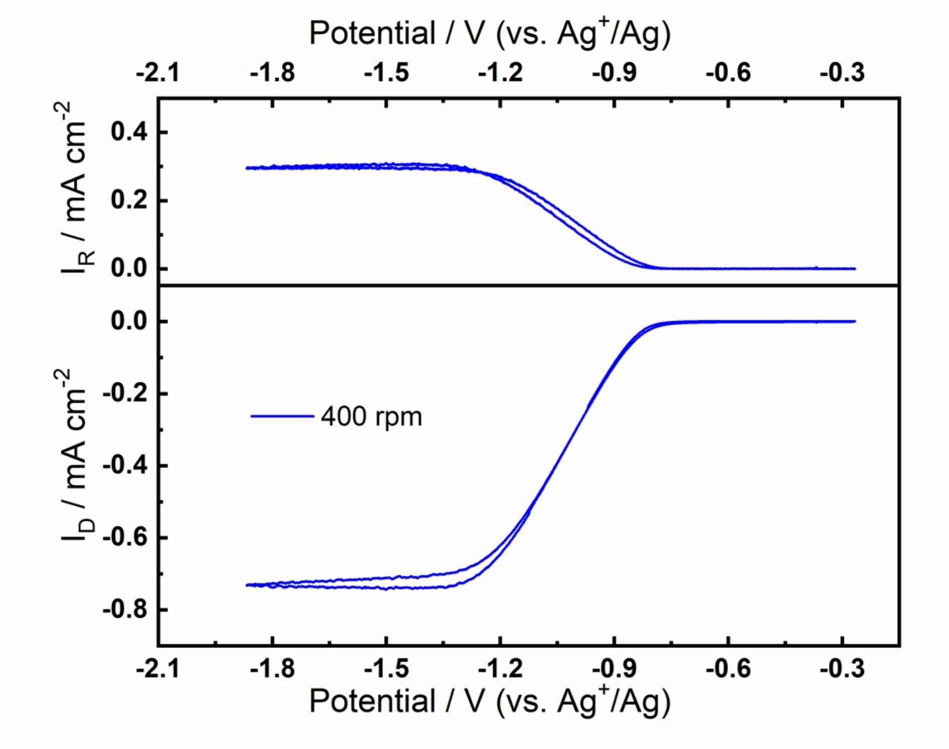

Figure S13. RRDE measurement at 100 mV s^{-1} at 400 rpm in 0.1 M $TBAClO_4$/DMSO. The potential of ring is -0.37 V.

In Ca^{2+}-free electrolytes, the superoxide (TBA^+-associated O_2^-) generated during the ORR is soluble in the electrolyte and thus can be detected by the ring electrode under forced convection.

Well-defined limiting currents can be obtained on both the disc and ring electrodes during the ORR, suggesting a diffusion-controlled behaviour. From the ratio of ring/disc current densities, collection efficiency of the used RRDE can be determined to be 41%, which will be applied in **Figure S14**.

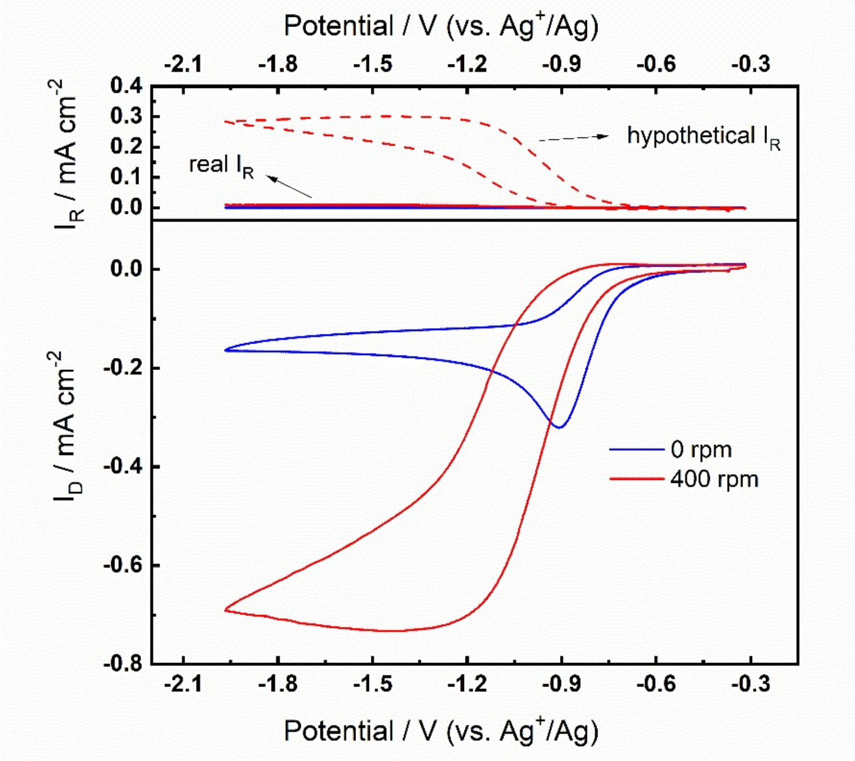

Figure S14. RRDE measurements at 100 mV s^{-1} at 0 rpm and 400 rpm in 0.1 M $TBAClO_4$/ 0.01 M $Ca(ClO_4)_2$/DMSO. The dashed line is the hypothetical ring current expected for complete superoxide formation without any other products generated in the ORR. The ring current data (denoted as real I_R) taken from **Figure 7(a)** is overlaid for comparison.

The hypothetical ring current is calculated by the following equation, where I_D is the obtained disc current and 0.41 is the collection efficiency of RRDE determined from data in **Figure S13**.

$$I_R(hypothetical) = I_D \times 0.41$$

From **Figure S14**, if the disc current response were to be completely due to the formation of soluble superoxide, the obtained ring current should be much larger than what we have observed. Therefore, the RRDE results suggest that soluble superoxide only takes up a small proportion (*ca.* 3%, estimated from the ratio of real I_R to hypothetical I_R), and most of the products remain confined at the electrode interface and leads to passivation.

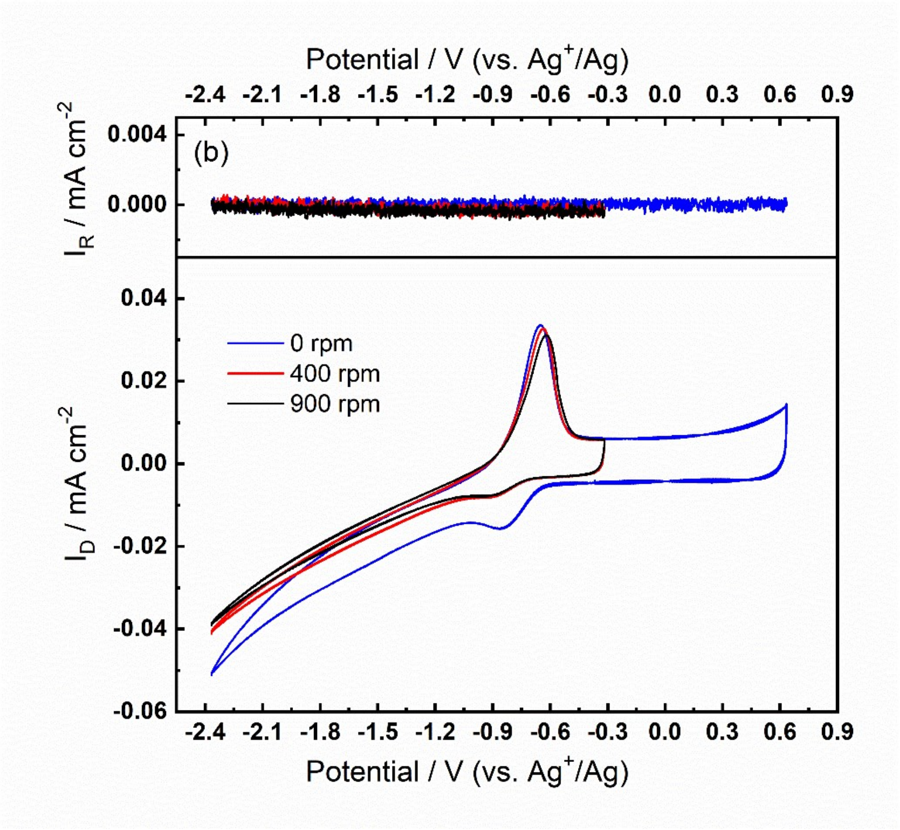

Figure S15. RRDE measurements at 100 mV s^{-1} at 0 rpm, 400 rpm, and 900 rpm after the oxidation peak appears in 0.1 M $TBAClO_4$/0.01 M $Ca(ClO_4)_2$/DMSO. The potential of ring is -0.37 V. The data for 400 rpm and 0 rpm are taken from **Figure 7(b)** for comparison.
